# Longitudinal unzipping of 2D transition metal dichalcogenides

**DOI:** 10.1038/s41467-020-18810-0

**Published:** 2020-10-06

**Authors:** Suchithra Padmajan Sasikala, Yashpal Singh, Li Bing, Taeyoung Yun, Sung Hwan Koo, Yousung Jung, Sang Ouk Kim

**Affiliations:** 1 National Creative Research Initiative Centre for Multi-Dimensional Directed Nanoscale Assembly, Department of Materials Science and Engineering, KAIST, Daejeon, 34141 Republic of Korea; 2 Graduate School of EEWS, KAIST, Daejeon, 34141 Republic of Korea

**Keywords:** Electrocatalysis, Electrocatalysis

## Abstract

Unzipping of the basal plane offers a valuable pathway to uniquely control the material chemistry of 2D structures. Nonetheless, reliable unzipping has been reported only for graphene and phosphorene thus far. The single elemental nature of those materials allows a straightforward understanding of the chemical reaction and property modulation involved with such geometric transformations. Here we report spontaneous linear ordered unzipping of bi-elemental 2D MX_2_ transition metal chalcogenides as a general route to synthesize 1D nanoribbon structures. The strained metallic phase (1T′) of MX_2_ undergoes highly specific longitudinal unzipping owing to the self-linearized oxygenation at chalcogenides. Stable dispersions of 1T′ MoS_2_ nanoribbons with widths of 10–120 nm and lengths up to ~4 µm are produced in water. Edge abundant 1T′ MoS_2_ nanoribbons reveal the hidden potential of idealized electrocatalysis for hydrogen evolution reactions at a competitive level with the precious Pt catalyst.

## Abstract (web summary)

Reliable unzipping of the basal plane of mono-elemental graphene and phosphorene has only been reported thus far. Here, the authors demonstrate the unzipping of bi-elemental 2D MX_2_ transition metal chalcogenides as a general route to synthesize 1D nanoribbon structures.

## Introduction

Discovery of 2D atomic structures, including graphene, transition metallic dichalcogenides (TMDs), *h*-boron nitride, phospherene, and mxene, has unveiled new possibilities in materials science^1^. Unzipping of the basal plane is a general issue to uniquely control the material signatures of the 2D materials, as evidenced by the effective transformation of intrinsically metallic graphene into semiconductors, while unzipped into few-nanometer-wide graphene nanoribbons^2^. Chemical modification of abundant edge sites endowed from the geometric transformation as well as quantum state confinement provides generic opportunities towards the modulation of diverse physicochemical characteristics^3^. Despite the well-recognized advantages, however, reliable unzipping has been successful only for graphene and phosphorene thus far^2,4,5^.

2D TMDs (MX_2_) are a unique family of bi-elemental compounds consisting of an atomic layer of transition metal (M = Mo/W) sandwiched between chalcogenide elements (X = S/Se/Te)^6^. Motivated from the prediction of ideally balanced binding characteristics for hydrogen, MX_2_ is particularly promising for the electrocatalytic hydrogen evolution reaction (HER) without the use of precious metal catalysts^7^. Unfortunately, the abundant crystalline basal planes of MX_2_ are catalytically inert and the HER activity principally originates from their unsaturated edges and defects. While the edge to surface ratio of 2D MX_2_ is highly limited, a considerable amount of research efforts has been devoted to promote the active sites by the shape engineering of mesoporous defect-rich films and vertical nanoflakes, or heteroelement doping^8–12^. Alternatively, phase engineering by Li ion intercalation has been found to induce a transition from intrinsic semiconducting phase (trigonal prismatic, 2H) to strained metallic phase (distorted octahedral, 1T′), which can also enhance the HER activity accompanied by the increased electron mobility^13^. Nevertheless, in contrast with noble metal catalysts such as Pt nanoparticles, the electronic structure of atomically thin materials is strongly influenced by local electrochemical reactions. Molecular absorption during HER at MX_2_ surface can dramatically reduce the conductivity of MX_2,_ which can subsequently slow down the reaction kinetics and reduce the overall current density^14^ Thus, increasing the edge sites is essential for the optimal catalytic performance. Unfortunately, the intrinsic three-atom-thick nature and multi-elemental crystalline structures of 2D MX_2_ poses challenges for the delicate shape engineering towards desired morphologies and structures^15–17^. Here, we introduce solution-phase unzipping of 2D MX_2_ under aqueous condition as a general route to the production of edge-enhanced MX_2_ nanoribbons (NRs).

## Results

### Synthesis and structural characterization of MX_2_ nanoribbons

**Fig. 1:**
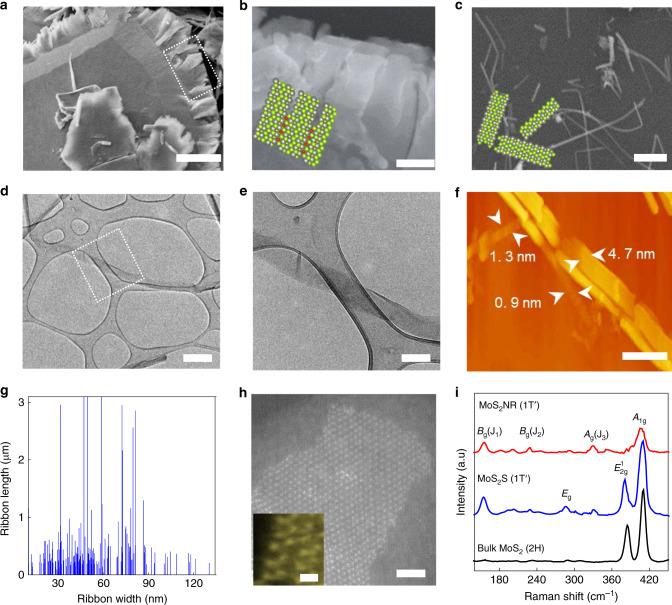
Unzipping of 2D MX_2_. SEM image of (**a**) unzipped MoS_2_ after 5 min of ultrasonication displaying the initiation of unzipping from flake edge, (**b**) Magnified image for unzipped edge and (**c**) Fully unzipped MoS_2_ displaying discrete MoS_2_ NRs. Scale bars, (**a**, **c**) 0.5 and (**b**) 0.1 µm. **d**, **e** TEM image of isolated MoS_2_ NRs. Scale bars, (**d**) 0.2 and (**e**) 0.1 µm (**f**) AFM image of MoS_2_ NRs. Scale bar, 0.2 µm (**g**) Plot of NR length as a function of width measured by TEM of more than 400 MoS_2_ NRs. **h** STEM image of MoS_2_ NR. Inset is the lattice image with low beam exposure displaying 2a0xa0 symmetry and Mo terminated zigzag edge structure. Scale bars, 1.5 and 0.15 nm (inset). **i** Raman spectra of bulk MoS_2_, 1T’ MoS_2_ sheets (MoS_2_ S) and MoS_2_ NRs.

Our unzipping is a two-step process consisting of the intercalation of bulk MX_2_ with Li^+^ ions (to obtain Li_x_MX_2_) and subsequent ultrasonication in oxygenated water to form a stable suspension. Upon the ultrasonication, Li_x_MX_2_ undergoes spontaneous exfoliation and longitudinal unzipping (more details are included in Methods section, Supplementary Fig. 1). The generality of this method has established with different 2D MX_2_ samples, including MoS_2_, MoSe_2_, MoTe_2,_ and WSe_2_ (Fig. 1 and Supplementary Figs. 2–4). Scanning electron microscopy (SEM) image presents the initiation of unzipping from the edge of bulk MoS_2_ crystal (Fig. 1a, b). A further propagation of the directional unzipping under ultrasonication creates fully unzipped MoS_2_ nanoribbons (NRs) together with minor portions of residual nanosheets and layered flakes (Fig. 1c and Supplementary Fig. 5).

The NRs showed a great colloidal stability in aqueous media. It has been established from previous reports that chemically exfoliated MoS_2_ dispersion in water has negatively charged MoS_2_ layers with lithium cations and hydroxide anions (Li^+^+(MoS_2_)^x-^+(1-x) OH^−^)_aq_. The range of negative charge has been reported to be 0.15–0.25 per Mo atom, which ensures a great colloidal stability in aqueous media^18,19^. The discrete NRs showed an average length of 0.3 µm even though occasionally length up to 4 µm can also be seen. The width of NRs ranged from 10 to 120 nm (Fig. 1c–g, Supplementary Figs. 6 and 7). AFM measurement of MoS_2_ NRs displayed thickness of 0.7–4.7 nm (Fig. 1f, Supplementary Fig. 5C). Detailed investigation on the crystalline structures of MoS_2_ NR verified 1T′ crystal lattice structures with Mo-zig zag edge structure (Fig. 1h and Supplementary Fig. 8). Notably, the 1T′ phase is unstable under electron beam exposure and thereby get relaxed to 1T and 2H structure under electron microscopy imaging (Supplementary Fig. 9)^20^.

Raman spectrum of the MoS_2_ NR displayed signature peaks for 1T′ phase at 156 (J_1_), 228 (J_2_) and 330 cm^−1^ (J_3_) (Fig. 1i)^21,22^. Interestingly, the peak for in-plane vibration mode at 383 cm^−1^ (E^1^_2g_) is absent in the spectrum, indicating that out-of-plane vibration mode at 405.5 eV (A_1g_) is more excited, which indeed confirms the high density of edge sites^23^. Similar Raman spectra is observed for NRs prepared from other MX_2_ samples (Supplementary Fig. 2–4).

Additional evidence for the metallic nature of 1T′ MoS_2_ NRs is obtained from four-probe hall-effect measurements. A thin film of NRs spin coated on Si/SiO_2_ substrate showed resistivity of 1.68 × 10^−2^ Ωcm (Supplementary Fig. 10A, B). The resistivity of MoS_2_ NRs is comparable to the 1T′ MoS_2_ nanosheets prepared by hydrothermal synthesis (10^−1^ Ωcm) and chemical exfoliation (10^1^–10^−2^ Ωcm) but higher than those prepared by solid-state reaction (10^−3^ Ωcm) and exfoliation with high-power forming-gas microwave plasma (8.6 × 10^−5^ Ωcm)^24–28^. The relatively low electron mobility of MoS_2_ NRs (2.7 × 10^−2^ cm^2^ V^−1^ s^−1^) suggests the presence of defects and vacancies as well as edge sites^6,29^. The linear I-V characteristics of MoS_2_ NRs advocate ohmic behavior (Supplementary Fig. 10C). The local electronic properties of a single MoS_2_ NR is obtained by employing conductive AFM (c-AFM) measurement (Supplementary Fig. 10D). The I-V measurement of an individual MoS_2_ NR showed ohmic behavior (Supplementary Fig. 10E). However, the current map image showed inconsistent current density on the NR with a scan rate of 0.7 Hz and applied voltage of 200 mV (Supplementary Fig. 10F) indicating the instability of metallic 1T′ phase as evident from the STEM measurement (Supplementary Fig. 9).

Thermal annealing can induce the phase transformation of MoS_2_ NRs from metallic to semiconductor phase (Supplementary Fig. 11). The transformation progresses gradually upon a thermal annealing above 100 °C and almost completes at 400 °C. The 2H phase contribution has been increased to more than 93 ± 3% at 400 °C. Notably, a peak around 236 eV, representative of Mo^6+^ 3d_5/2_, is absent in the XPS spectra indicating that the oxidation of Mo is minimal in the all samples (Supplementary Fig. 11A). In addition, quenching of J_1_ and J_2_ and J_3_ peaks is observed in the Raman spectra for the samples annealed above 100 °C (Supplementary Fig. 11B). A_1g_ peak becomes increasingly prominent and in-plane vibration modes at 383 cm^−1^ (E^1^_2g_) has been restored with annealing temperature. The characteristic MoS_2_ excitonic A and B peaks (600–700 nm) and a sharp convoluted C&D excitonic peak (~420 nm) have gradually emerged as a function of annealing temperature in the absorption spectra, indication of phase change (Supplementary Fig. 11C). An emergence of weak PL peak was detected around 656 nm in the photoluminance spectrum (Supplementary Fig. 11D) of MoS_2_ NRs annealed at 400 °C, which also supports the formation of semiconducting 2H phase^26^.

### Mechanism of MX_2_ unzipping

According to previous literature, oxygenation at the basal plane of MX_2_ upon ambient condition has been reported not to be site-specific but randomized, and thereby generally leads to the etching in a disordered manner to yield random porous MX_2_ structures^30,31^. Noteworthy that those previous works have focused on the 2H phase of MX_2_. As a control test, we repeated our experiment with the MoS_2_ samples of pure 2H phase and mixed phase of 1T′ and 2H (Methods). The obtained exfoliated sheets are randomly etched and highly porous in nature in accordance with the previous works in contrast to longitudinal unzipped structures obtained from pure 1T′MoS_2_ (Supplementary Fig. 12). Typical synthesis methods for 1T′MX_2_ results in a mixture of 2H and 1T′ phase. However our optimized lithium intercalation method yield more than 90% 1T′ phase, which is found to be very critical for their basal plane unzipping (associated discussion is given in Supplementary notes and Supplementary Figs. 13 and 14)^7,32^.

**Fig. 2:**
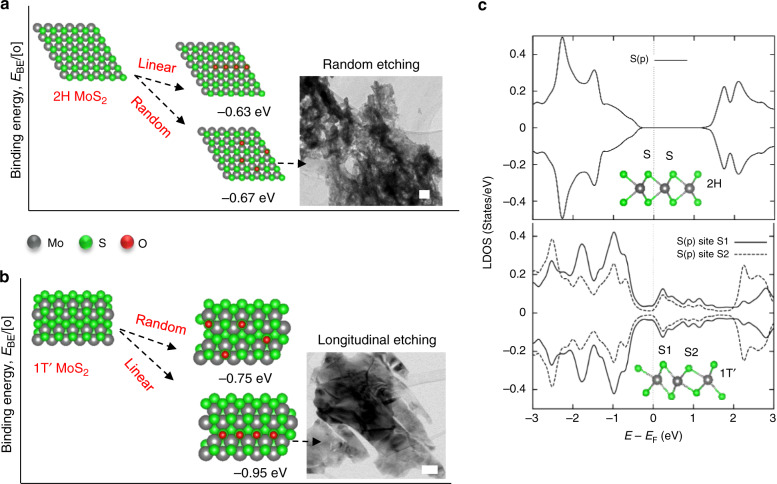
Oxygenation on the surface of MX_2_. **a**, **b** Difference in oxygen binding energies at 2H and 1T′ MoS_2_. While 2H phase prefers random binding of oxygen at surface, 1T′ phase prefers a linearized pattern. Insets show TEM images of randomly and linearly etched MoS_2_ sheets. Scale bars, 100 nm. **c** The local density of states diagrams representing the similar (top) and different (bottom) density of states for S atoms in 2H and 1T′ phases.

We have performed density functional theory (DFT) calculation and compared the oxygen binding energy at the surface of 1T′ and 2H phases (Fig. 2a, b and Supplementary Fig. 15A, B) for a further understanding of the unzipping mechanism in different crystalline phases. Binding energy per oxygen atom (E_BE_) is calculated by the equation; E_BE_ = (E_*O_-(E_*_ + nµ_O_))/n, where E_*_ and E_*O_ denotes the energy of MX_2_ with and without O atoms. µ_O_ is the chemical potential of O atom evaluated as ½ µ_O2_. Comparison of the E_BE_ values at 1T′MX_2_ surfaces for linear ordered vs. random arranged ones suggests a thermodynamic tendency towards a linearly ordered oxygenation (Table 1). By contrast, all 2H phases of MX_2_ showed lower E_BE_ values for random site oxygenation, in an agreement with the typical randomized etching. Investigation on such a preference for the longitudinal ordering in 1T′ phase by means of spin-resolved local density of states analysis showed the existence of two different types of X (S/Se/Te) sites (S1 and S2) with different electron densities (Fig. 2c and Supplementary Fig. 15C–E). The S1 sites have a higher density of states at fermi level, and hence a stronger hybridization tendency can be anticipated between the *p* orbitals of X and oxygen compared to the S2 sites with a lower density of states. In the case of 2H phase, oxygen binding is randomized since such an asymmetry is absent (Fig. 2c).

**Table 1:** Phase-dependent oxygenation.

System	2H phase		1T′ phase	
	Random	Linear	Random	Linear
MoS_2_	−0.67	−0.63	−0.75	−0.95
MoSe_2_	0.63	0.95	1.01	−0.85
MoTe_2_	−0.43	0.23	0.63	−1.80
WSe_2_	0.31	0.41	0.36	−0.11

Oxygen binding energies (ΔE/O atom) for different 2D MX_2_ phases in the unit of eV.

**Fig. 3:**
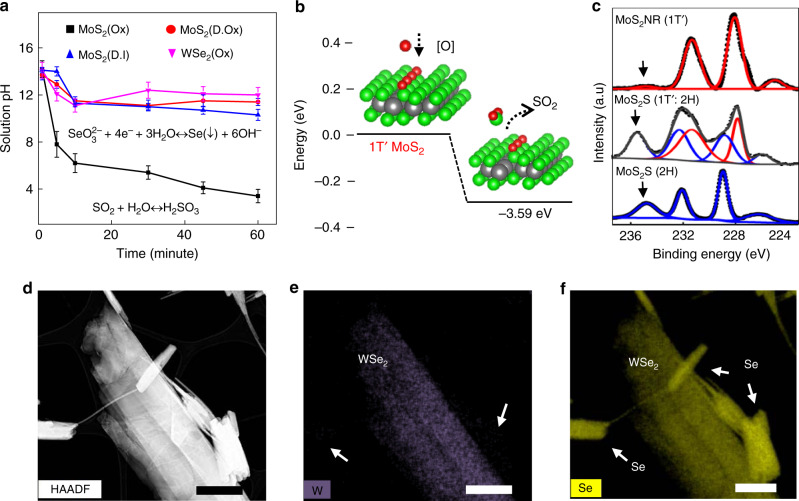
Mechanism for longitudinal unzipping. **a** Solution pH changes upon the ultrasonication of Li intercalated MoS_2_ in oxygenated (Ox), deoxygenated (D.Ox), and deionized (D.I) water. Solution pH change upon hydration of Li intercalated WSe_2_ in oxygenated water is also provided. **b** Schematic presentation of energetically favored removal of SO_2_ from MoS_2_ surface. **c** XPS analyses of Mo3d orbits of MoS_2_ sheets of pure 2H phase and 2H:1 T′ combined phase and MoS_2_ NRs prepared in oxygenated water. **d**–**f** HAADF and Elemental mapping (W, and Se) of partially unzipped WSe_2_ layered sheet displaying Se as a byproduct and line defects on the basal plane. Scale bars, 0.2 µm.

A strong alkaline pH is generally expected for the hydration of lithium intercalated MX_2_ due to the generation of LiOH. Interestingly, the pH of the exfoliated solution drops drastically from alkaline (pH = 14.2) to acidic (pH = 3.4) upon the ultrasonication of Li_x_MoS_2_ in oxygenated water (Fig. 3a). We infer that the rapid decrease of pH upon ultrasonication should result from the dissolution of SO_2_ released from the unzipping reaction (SO_2_ + H_2_O ↔ H_2_SO_3_). We evaluated the energies associated with the evolution of SO_2_/SeO_2_/TeO_2_ species upon creating S/Se/Te vacancies in the basal plane of 1T′ phase to assess a simple thermodynamic aspect of this reaction route. All the formation energies were found to be negative (−3.59, −1.90, −1.20, and −1.60 eV for MoS_2,_ MoSe_2_, MoTe_2,_ and WSe_2_, respectively) indicative of the feasibility (Fig. 3b). The longitudinal removal of XO_2_ (SO_2_/SeO_2_/TeO_2_) can be considered responsible for relatively low oxygen content in the resultant MX_2_ NRs compared to etched 2H and mixed-phase MX_2_ nanosheets (Fig. 3c and Supplementary Fig. 2–4D). In addition, line faults are observed in SEM images of hydrated Li_x_MoS_2_ (Supplementary Fig. 16A–F). Interestingly, pH of the exfoliated solution did not show any significant drop upon ultrasonication of Li_x_WSe_2_ in oxygenated water (Fig. 3a). While scanning transmission electron microscopy (STEM) observation of partially unzipped MoS_2_ identifies only MoS_2_ (Supplementary Fig. 16D–F), those of WSe_2_ detects the by-product of Se (Fig. 3d–f). This observation further confirms the evolution of XO_2_ from the MX_2_ surface since SeO_3_^2−^ yielded from the dissolution of SeO_2_ in water is known to undergo crystallization in basic conditions (SeO_3_^2−^+4e^−^+3H_2_O ↔ Se(↓)+6OH^−^).

Notably, pH change is not prominent while Li_x_MoS_2_ was ultrasonicated in deoxygenated or deionized water (Fig. 3a). In addition, we did not observe any significant unzipping when 1T′ MoS_2_ nanosheets (obtained by hydration of Li_x_MoS_2_ in deoxygenated water) further ultrasonicated in oxygenated water. These results imply that the exothermic hydration of Li^+^ supports the kinetics of XO_2_ removal. A high concentration of dissolved oxygen can accelerate the removal of SO_2_ creating chalcogenide vacancies during the hydration of Li_x_MoS_2_ in water. Taken together, the underlying mechanism for the NR formation can be suggested as chemo-mechanical unzipping of MX_2_ through the line faults created by chalcogenide vacancies under the sonochemical agitation of hot gas bubbles.

### Hydrogen evolution reaction performance of nanoribbons

**Fig. 4:**
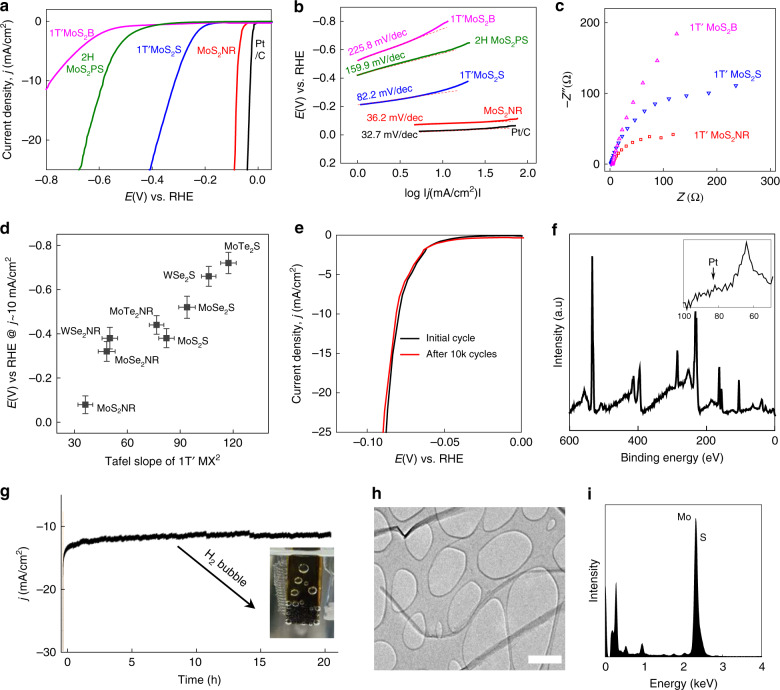
HER electrocatalytic activity of MX_2_ NRs. **a** Linear sweep voltammograms and (**b**) corresponding Tafel plots for MoS_2_ NR, 1T′ MoS_2_ sheet (1 T′ MoS_2_S), 2H MoS_2_ porous sheets (2H MoS_2_PS) and bulk 1T′ MoS_2_ (1 T′ MoS_2_B). Pt/C is included as reference. **c** Electrochemical impedance spectroscopy data for MoS_2_ NR, 1T′ MoS_2_S, and 1T′ MoS_2_B. **d** Tafel slopes and required potentials to attain a current density of 10 mA/cm^2^ for all unzipped 1T′ MX_2_ NRs, compared to exfoliated 1T′ MX_2_S. **e** Polarization curves for MoS_2_ NR before and after 10,000 CV scans. **f** XPS spectrum showing no Pt contamination during electrocatalytic cycling, inset is zoomed for 50–100 eV. **g** Chronoamperometric response of MoS_2_ NR at an applied potential of 80 mV vs. RHE displaying variation of current density up to 20 h. Inset shows photograph of H_2_ bubbling on the electrode surface (**h**) TEM of MoS_2_ NR catalyst after10000 cycles. Scale bar, 0.5 µm. **i** Associated EDS spectrum.

We have investigated the HER catalytic activity of MX_2_ samples using a standard three-electrode electrochemical configuration in 0.5 M H_2_SO_4_ electrolyte (Fig. 4). In order to investigate significance of unzipping, we compare HER activity of various MoS_2_ samples. Figure 4a, b present the polarization curves and Tafel plots of MoS_2_ samples, including 1D MoS_2_ NRs unzipped from 1T′MoS_2_ (MoS_2_ NR), 2D 1T′MoS_2_ sheets (1T′MoS_2_S), unexfoliated bulk 1T′MoS_2_ (1T′MoS_2_B) and liquid phase exfoliated 2H MoS_2_ porous sheets (2HMoS_2_ PS). MoS_2_ NR exhibits the highest HER performance even with a small loading (15 µg/cm^2^), while attaining a current density (*j*) of 10 mA/cm^2^ at very low potential (~79 mV versus RHE). Tafel slope and exchange current density is measured to be 36.2 mV/dec and 8.8 × 10^−6^ A/cm^2^, respectively. 1T′MoS_2_S showed a better HER performance than 1T′MoS_2_ B, verifying the significance of exfoliation of layered MX_2_ for catalysis. Interestingly, 2HMoS_2_ PS showed the lowest HER performance among all samples. Even with the high density of edge sites decorating random porous morphology, the low electrical conductivity of 2H phase is a bottleneck for the overall electrocatalytic activity. Noteworthy that MoS_2_ NR showed lower resistance in the electrochemical impedance spectrum than 1T′ sheet due to the high charge transfer from the edge sites (Fig. 4c). The annealed MoS_2_ NRs showed a lower HER activity compared to 1T′MoS_2_ NRs (Supplementary Fig. 17A). This is in agreement with previous studies, which showed higher HER activity of 1T′ phase MoS_2_ to corresponding 2H phase MoS_2_ samples^33,34^. DFT studies have indicated that even though the free energy of hydrogen absorption of Mo-edge of 2H-MoS_2_ is comparable to that of 1T′-MoS_2_, the kinetics of HER is restricted due to poor charge transfer in semiconducting 2H-MoS_2_ leading to lower HER activity^35^.

Indeed, the optimal integration of dense edge active sites with metallic 1T′ phase is unambiguously demonstrated by the excellent electrocatalytic HER kinetics for all MX_2_ NRs (Fig. 4d, Supplementary Fig. 17B–E). A comparison of electrochemical activities among the previously reported 2D MX_2_ catalysts and our unzipped MX_2_ is provided (Supplementary Table 1). Another critical feature for the viable HER catalyst is electrochemical stability. The HER polarization curve of MoS_2_ NR displays no significant modification (ΔV = 3 mV @ 25 mA/cm^2^) after a 10,000 cycling test, which verifies a long-term durability (Fig. 4e). Notably, we did not observe any metal contamination even after 10,000 cycles (Fig. 4f).

MoS_2_ NR shows a highly stable current density under continuous operation at 80 mV for 20 h (Fig. 4g). Post catalysis characterization suggests that MoS_2_ NRs can endure the long-term electrochemical stressing, as evidenced by the morphology or chemical state characterization (Fig. 4h, i). The NRs displayed wrinkled morphology and slight aggregation, presumably due to the residual nafion contamination during electrode preparation, but well-maintained the 1T′ crystal structure (Supplementary Fig. 18).

We calculated the turnover frequency (TOF) to compare the intrinsic activity among various MX_2_ NRs based on the assumption that only edge sites of MX_2_ NRs are active for HER (Supplementary notes)^36^. After normalization with the typical active site density of Pt (1.5 × 10^15^ sites/cm^2^), we have calculated TOF of MoS_2_ NRs to be 3.43 s^−1^, higher than any previously reported molybdenum sulfide catalysts, including amorphous cluster (0.07 s^−1^), nanoparticles (0.02 s^−1^), vertical flakes (0.01 s^−1^), and sulfur vacant strained MoS_x_ (0.31 s^−1^)^9,36–38^. Such a high TOF value of MoS_2_ NR implies the existence of optimal catalytic sites even higher than Pt(III) surface (0.94 s^−1^)^36^. Other MX_2_ NRs also showed favorable TOF values, including 0.017 s^−1^ for MoSe_2_, 0.142 s^−1^ for MoTe_2,_ 0.006 s^−1^ for WSe_2_ (Supplementary Table 2). The high HER activity of MoS_2_ NRs can be supported by the electrochemically active surface areas (ECSA), which were calculated from the double-layer capacitance (C_dl_) obtained by cyclic voltammetry curves (Supplementary Fig. 19). Considering that ECSA is directly proportional to capacitance, MoS_2_ NRs with a high C_dl_ (10.1 mF/cm^2^) attain the highest ESCA among the MX_2_ NRs investigated in this study (Supplementary notes and Supplementary Table 1).

**Fig. 5:**
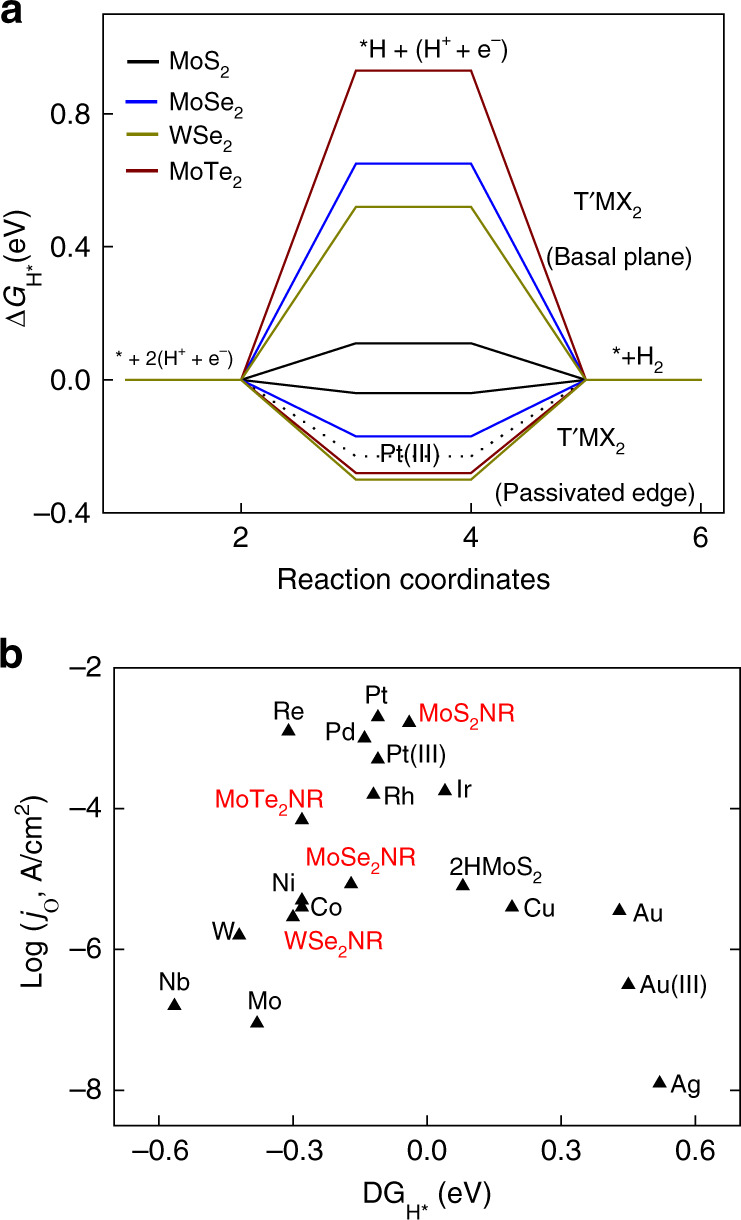
Theoretical investigation of HER of MX_2_ NRs. **a** Calculated Gibbs free energy diagram for hydrogen binding at the basal plane and edge sites of 1T′ MX_2_ systems at equilibrium potential (U = 0 V). **b** Volcano plot of the exchange current density vs. free energy for atomic hydrogen binding at MX_2_ NRs and pure metal catalysts. Exchange current density of MX_2_ NRs is normalized with the atomic site density of Pt for comparison.

It is noteworthy that while edges play leading contribution, other factors, such as basal-plane activity should also have contributed to the overall activity of MX_2_ NRs. Accordingly, we evaluated the HER activity of 1T′MX_2_ using DFT by comparing the Gibbs free energy (ΔG_H*_) for hydrogen adsorption at both basal plane and edge active sites. The calculation successfully predicts the highly active sites at the passivated edges of 1T′MX_2_ compared to basal plane (Fig. 5a). The ΔG_H*_ close to zero at MoS_2_ edge site (−0.04 eV) validates the highest HER activity of MoS_2_ NR among all tested MX_2_ NRs in this study. This is attributed to the relative electron deficiency of Mo sites arising from the stronger electronegativity of neighboring S atoms compared to other chalcogenides. For a quantitative comparison with the typical metal catalysts, a Volcano plot is prepared with the experimental exchange current density plotted against the DFT calculated ΔG_H*_^36,39^. While all MX_2_ NRs well-follow the volcano trend, MoS_2_ NR occupies the desirable position comparable to those of precious Pt group metals (Fig. 5b).

## Discussion

We have demonstrated with the help of DFT studies a phase-dependent oxygenation pattern in 2D MX_2_. While 2H phase MX_2_ undergoes oxygenation at random positions, the oxygenation in 1T′ MX_2_ is linear ordered due to the presence of two types of chalcogenides with different electron densities. In contrast to typical chemisorption, chalcogen atoms are removed in the form of XO_2_ (SO_2_, SeO_2_, and TeO_2_) during the oxygenation process creating line faults under the exothermic hydration of Li_x_MX_2_ in oxygenated water. Sonochemistry and agitation of ultra-hot gas bubbles involved in the sonication cause the chemo-mechanical unzipping of 1T′ MX_2,_ which initiates and propagates through these line faults. Abundant active edges decorating the 1T′ NRs serve for the well-balanced molecular binding sites for optimal HER catalyst. This effective geometric transformation exploiting delicate surface reactivity control highlights how to tailor the nanoscale morphology of polymorphs of low-dimensional multi-element structures. Thus, a broad spectrum of potential functionalities depending on diverse choices of chemical compositions can be extrapolated.

## Methods

### Synthesis of lithium intercalated MX_2_ (LixMX_2_)

Bulk MX_2_ (MoS_2_, WSe_2_, and MoSe_2_) flakes of 99.8% purity were purchased from Alfa Aesar. MoTe_2_ crystals were purchased from Sigma Aldrich and ground to powder. All samples were first degassed by keeping under vacuum at 60 °C for 48 h. Degassed MX_2_ (100 mg) was treated with different concentrations of *n*-butyl lithium (*n*-BuLi) (stock concentration of 1.6 M, Sigma Aldrich) in hexane at a fixed dose of 10 mg/ml under different temperature from 20 to 120 °C. The treatment time was varied from ~6 to 100 h to synthesize Li_x_MX_2_. After a predetermined intercalation time, the suspension was filtered over Millipore membrane and washed several times with high purity hexane to separate the intercalated MX_2_ from residual *n*-BuLi. The entire procedure was carried out in a glove box under extra pure N_2_ atmosphere. Since lithium compounds react violently in the presence of humid air, dealing with a large amount of Lithium compounds requires cautious treatment. We have made several small batches (2 ml) inside the N_2_-filled glove box to acquire a sufficient amount of materials for this study.

### Synthesis of 2D MX_2_ sheets (MX_2_S)

To prepare MX_2_S sheets of pure 1T′ and 1T′:2H combination phase (associated discussion is in Supplementary file and supplementary Figs. 13 and 14), purified Li_x_MX_2_ was exfoliated in deionized water by ultrasonication for 2 h. The solution was then slowly dropped into a hexane column (3000 ml) at a rate of 0.1 ml/minute to wash away any organic residues. The recovered solution from the hexane column is then centrifuged several times in high purity deionized water to remove the impurities and unexfoliated MX_2_. The aliquot was recovered to obtain exfoliated MX_2_ sheets. To prepare unexfoliated bulk 1T′ MX_2_ (1 T′ MX_2_ B), purified Li_x_MX_2_ was hydrated in deionized water without ultrasonication to prevent exfoliation. Pure 2H phase exfoliated MX_2_ sheets (2HMX_2_S) were obtained by liquid phase exfoliation. In a typical procedure, bulk MX_2_ (1 mg/ml) was ultrasonicated in a mixture of ethanol-water (1:1; v;v) for 1 h and centrifuged to recover the aliquot containing exfoliated 2HMX_2_ sheets. The exfoliated MX_2_ sheets in ethanol water mixture is solvent exchanged to oxygenated water by further centrifugation and redispersion procedures. This is then ultrasonicated for 2 h to investigate the oxygenation (2H MX_2_PS). Oxygenated water was prepared by purging O_2_ gas of 99.9% purity directly into 200 ml of deionized water in a closed glass vessel for 1 h. In order to prepare the deoxygenated water, 200 ml of deionized water was placed in a stoppered Buchner flask attached to a vacuum pump. The water was heated to 60 °C with magnetic stirring for 1 h under vacuum to get rid of all the dissolved oxygen in the deionized water.

### Unzipping of MX_2_

After intercalation reaction with optimized conditions to achieve pure 1T′ phase, the recovered Li_x_MX_2_ was transferred to a vessel containing oxygenated water. The suspension was ultrasonicated and dropped into a hexane column (3000 ml) at a rate of 0.1 ml/min to wash away *n*-BuLi residue. Afterwards, the recovered suspension of MX_2_ in water from the bottom of the hexane column was further diluted in oxygenated water (300 ml) and ultrasonicated for 2 h. The suspension was aged sufficiently before centrifugal separation (20,000 rpm for 2 h) to recover the unzipped MX_2._ The obtained MX_2_ NRs was redispersed in ethanol or water and centrifuged several times from 8000 to 3000 rpm, each time discarding the sediments to remove the unexfoliated and partially unzipped MX_2_. Noteworthy that only ~2.0 ± 1.4% of the starting material was retained in the supernatant. The majority of raw material was etched away and/or remained as bulk layered structures that could be removed by centrifugation. We found that a prolonged sonication does not lead to a higher yield of NRs. The sonication time was carefully controlled to ~2 h for the optimal yield of NRs. Note that, the experimental conditions described here such as initial Li ion concentration, temperature, reaction time, sonication time, and purification steps may change depending on the crystal quality and flake size of precursor source. The aliquot recovered after centrifugation was drop-casted on Cu-carbon grids for TEM and STEM measurements and Si/SiO_2_ substrate for XPS and Raman measurements. For HER electrode preparation, the aliquot was vacuum dried under N_2_ at 40 °C.

### Characterization of MX_2_ NRs

TEM, HRTEM, and EDS were performed with field emission TEM of 200 kV (Talos F200x, Tecnai F20, FEI). HAADF-STEM characterization was carried out with a double-aberration corrected HRTEM (Titan G2 60–300, FEI) at 80 and 200 kV accelerating voltage. SEM imaging was obtained with ultra-high resolution field emission scanning electron microscope (Hitachi, SU8230). Optical microscope (Olympus, Japan) was used for the optical imaging. Raman and XPS spectra were recorded with ARAMIS (Horiba Jobin Yvon) and k-alpha (Thermo VG Scientific), respectively. Hall Effect measurements and I-V curves were obtained using Ecopia Hall Effect measurement system (HMS-3000) equipped with spring clipboard sample mounting boards (SPCB) containing four gold plated pins. NRs were spin coated on the Si/SiO_2_ substrates and directly mounted on the SPCB. The distance among pins was fixed as 5 mm. Silver paste was used to make a good ohmic contact between the pin and the sample. Conductive AFM (MFP-3D, Asylum Research) measurement was carried out with Cr/Pt-coated cantilevers (ConEG, Budget Sensors) and Cr/Au-coated Si substrate. A three-electrode electrochemical cell was used for the electrochemical measurements with a Bio-Logic, SP-200 potentiostat. MX_2_ deposited glassy carbon electrode acted as the working electrode with a Pt wire as the counter electrode and a saturated calomel electrode as a reference electrode. Known weights of MX_2_ samples (15 µg) dispersed in ethanol were directly deposited onto the working electrode using a micropipette and dried at 60 °C under N_2_ flow. 10 µl of 5% Nafion was deposited as a protective layer on the surface of sample placed on glassy carbon electrode. Any incidence of Pt contamination was ruled out by repeating the same experiments with carbon counter electrode. Calibration of the reference electrode to reversible hydrogen electrode potential (RHE) was conducted in 0.5 M H_2_SO_4_ electrolyte with Pt wire as both counter and working electrode and found to be E_RHE_ **=** E_SCE_ + 0.252 V. The electrolyte was constantly stirred by a magnetic stirrer during an electrochemical test to facilitate mass transport and prevent the accumulation of bubbles on the surface of electrode. Details of measurements and calculation of Tafel slope, ESCA, and TOF values are provided in the supplementary information.

### Computational details

Spin-polarized density functional theory (DFT) computations were performed using the Vienna Ab-initio Simulation Package (VASP) with the projector-augmented-wave (PAW) method to account for core-valence interactions. Details of calculations are provided in the supplementary information. STEM image simulations were obtained using an open-source software QSTEM^40^.

## Supplementary information

Supplementary Information

## Data Availability

Rest of the data that support the findings of this study are available from the corresponding author upon reasonable request. Source data are provided with this paper.

## Source data

Source Data
