# Author Correction: Longitudinal unzipping of 2D transition metal dichalcogenides

**DOI:** 10.1038/s41467-020-19388-3

**Published:** 2020-10-27

**Authors:** Suchithra Padmajan Sasikala, Yashpal Singh, Li Bing, Taeyoung Yun, Sung Hwan Koo, Yousung Jung, Sang Ouk Kim

**Affiliations:** 1 National Creative Research Initiative Centre for Multi-Dimensional Directed Nanoscale Assembly, Department of Materials Science and Engineering, KAIST, Daejeon, 34141 Republic of Korea; 2 Graduate School of EEWS, KAIST, Daejeon, 34141 Republic of Korea; 3 Department of Chemical and Biomolecular Engineering, KAIST, Daejeon, 34141 Republic of Korea

**Keywords:** Electrocatalysis, Electrocatalysis

Correction to: *Nature Communications* 10.1038/s41467-020-18810-0, published online 6 October 2020.

The original version of this Article contained an error in the author’s affiliations.

The affiliation of Yousung Jung with Department of Chemical and Biomolecular Engineering, KAIST, Daejeon 34141, Republic of Korea was inadvertently omitted. Yousung Jung was incorrectly associated with Graduate School of EEWS, KAIST, Daejeon 34141, Republic of Korea.

The original version of this Article omitted the following from the Acknowledgements:

“Y.S. acknowledges generous financial support from C1 Gas Refinery R&D Center (NRF- 2019M3D3A1A01069099).”

This has now been corrected in both the PDF and HTML versions of the Article.

