# Supplementary material for: Longitudinal unzipping of 2D transition metal dichalcogenides
Source: Nat Commun. 2020 Oct 6;11:5032. doi: 10.1038/s41467-020-18810-0 (PMC7538978; doi:10.1038/s41467-020-18810-0)
Supplement: Supplementary file 1 — Supplementary Information [file 41467_2020_18810_MOESM1_ESM.pdf]

Supplementary Information

Longitudinal Unzipping of 2D Transition Metal Dichalcogenides

Suchithra Padmajan Sasikala,[1] Yashpal Singh,[2] Li Bing,[1] Taeyoung Yun,[1] Sung Hwan Koo,[1] Yousung Jung,[2]* and Sang Ouk Kim[1]*

[1]National Creative Research Initiative Centre for Multi-Dimensional Directed Nanoscale Assembly, Department of Materials Science and Engineering, KAIST, Daejeon 34141, Republic of Korea.

[2] Graduate School of EEWS, KAIST, Daejeon 34141, Republic of Korea.

*Correspondence to: sangouk@kaist.ac.kr, ysjn@kaist.ac.kr

Supplementary Note 1. Synthesis of 1T′ MoS_2. Synthesis of pure 1T′ MX_2 is very important for applications entailing effective charge transfer and electrical conductivity. Currently available scalable synthesis methods for 1T′ MoS_2, including chemical exfoliation, electrochemical exfoliation, colloidal and hydrothermal synthesis, can produce only mixtures of 1T′ and 2H phase MoS_2. Chemical and electrochemical exfoliation methods facilitated by Li^+ intercalation with the typical maximum yields ~60-65% and ~40% of 1T′ phase, respectively[1-3]. We systematically investigated the evolution of 1T′ phase in the exfoliated MoS_2 sheets by optimizing different parameters, such as initial concentration of *n*-BuLi, intercalation time and temperature while keeping a fixed dose of precursor bulk MX_2 at 10 mg/ml (Supplementary Fig. 13A-D). After the intercalation reaction, all samples were subjected to similar purification procedure and exfoliation process by ultrasonication in 200 ml of deionized water. Upon initial optimization experiments with different *n*-BuLi concentrations (reaction time (24 h) and room temperature (20 °C) kept constant), we found that composition of IT′ phase in the exfoliated MoS_2 sheets increased initially but saturated around *n*-BuLi concentration of 0.48 M (Supplementary Fig. 13A). Afterwards, we kept 0.48 M as the optimal concentration of *n*-BuLi and investigated the effect of reaction temperature (Supplementary Fig. 13B). Interestingly, we found that the composition of 1T′ phase in MoS_2 increased up to 90 °C and a further increase in temperature was found to decrease the composition of 1T′ phase. For more insight on this behaviour, we have studied the effect of intercalation time at different temperatures. As shown in Supplementary Fig. 13C, while intercalation time did not have a significant effect at 20 °C, the composition of 1T′ phase increased steadily at 90 °C and reached a maximum of ~99% at 48 h. A further increase of intercalation time was found to adversely affect the evolution of 1T′ phase. From the results, we can assume that both high temperature and long exposure may cause the restoration of 2H phase due to probable desorption of intercalated Li ions. XPS spectra of Mo3d displays the gradual evolution of 1T′ phase (Supplementary Fig. 13D). Note that, the optimization parameters described here for achieving 1T′ phase may greatly depend on the flake size and crystal quality of bulk MoS_2.

Supplementary Note 2. Synthesis of 1T′ WSe_2. Typical wet chemical and chemical vapor deposition (CVD) synthetic methods for 2D WSe_2 have been reported to form 2H phase thus far[4,5]. Colloidal synthesis of 1T′ WSe_2 flower-like 3D nanostructures with an average diameter of 200 nm has been reported[6]. However, to the best of our knowledge, there is no report for the direct

production of 2D 1T′ WSe_2 sheets yet. Chemical exfoliation using organolithium intercalation treatment of bulk layered WSe_2 followed by exfoliation in water result in predominantly 2H phase WSe_2 sheets with a very small fraction of 1T′[7]. It is noteworthy that we have achieved 99.4% 1T′ phase of WSe_2 sheets by optimizing exfoliation parameters in this work. Supplementary Fig. 13E provides the result of initial optimization experiments carried out to investigate the intercalation assisted chemical exfoliation of bulk WSe_2 (2H phase) using different *n*-BuLi concentrations ranging from 0.064 M to 0.64 M. The composition of 1T′ phase in the exfoliated WSe_2 was found to increase up to 47% when the concentration of *n*-BuLi was 0.32 M. No significant improvement in the composition of 1T′ phase has been observed with a further increase of *n*-BuLi concentration after the 24 h of intercalation reaction. Li intercalation reaction at 40 °C was found to yield the highest composition of 1T′ phase in the exfoliated WSe_2 (82.6%) after the 24 h of reaction (Supplementary Fig. 13F). Kinetic experiments with different intercalation times showed that an increase of reaction time adversely affects the 1T′ phase at 40 °C, which yielded the maximum 1T′ phase of 98.7% after 12 h of reaction (Supplementary Fig. 13G), revealing the significance of reaction time, temperature and concentration in the phase change of WSe_2. XPS spectra of displaying the gradual evolution of 1T′ phase is provided in Supplementary Fig. 13H. Note that, the optimization parameters described here for achieving of 1T′ phase may greatly depend on the flake size and crystal quality of bulk WSe_2.

Supplementary Note 3. Synthesis of 1T′ $MoSe_2$ and $MoTe_2$. Chemical exfoliation of $MoSe_2$ nanosheets through *n*-BuLi treatment has been reported to form a mixture of 1T′ and 2H phases. The maximum 1T′-phase concentration achieved thus far is ~65%[2]. By contrast, chemical exfoliation method for $MoTe_2$ has not been reported yet. The synthesis of 1T′-$MoTe_2$ is mainly relying on chemical vapor deposition (CVD) or colloidal synthesis[8,9]. We have carefully optimized the reaction condition for chemical exfoliation in order to achieve maximized concentrations of 1T′ phase in $MoSe_2$ and $MoTe_2$. As shown in Supplementary Fig. 14A, the maximum yield of 1T′ phase (~55.3%) in the exfoliated $MoSe_2$ was obtained at an initial *n*-BuLi concentration of 0.32 M, after 24 h of reaction at 20 °C. In the case of $MoTe_2$, the maximum yield of 1T′ phase (~49.4%) was obtained at the *n*-BuLi concentration of 0.16 M at the same reaction temperature and time. Comparison of the exfoliation behaviors under different temperatures (Supplementary Fig. 14B) verified that the optimum temperature range of 50 and 60 °C is required for the maximum yield of

1T′ phase in the exfoliated $MoTe_2$ (~69.2 %) and $MoSe_2$ (~99%) sheets, respectively (24 h reaction with 0.32 and 0.16 M *n*-BuLi). Kinetic experiments with different intercalation times showed that while a reaction time of 24 h is required for the maximum yield of 1T′ phase in the exfoliated $MoSe_2$ (~99%), 6 h is required for $MoTe_2$ (~97.7%) sheets (Supplementary Fig. 14C). Note that, the optimization parameters described here for achieving of 1T′ phase may greatly depend on the flake size and crystal quality of bulk MX_2.

Supplementary Note 4. Electrochemical Characterization of MX_2 NRs. HER activity was measured using linear sweep voltammetry (LSV) between 0.1 and -0.5 V *vs*. RHE with a scan rate of 5 mV/s. Before LSV measurement, a cyclic voltammetry (CV) test for 10 cycles was performed at 50 mV/s to activate the working electrode. Impedance spectra measurement (EIS) was performed at a bias potential of -0.1 V *vs*. RHE, while sweeping the frequency from 1 MHz to 10 mHz with 5 mV AC amplitude. Chronoamperometric (CA) characterization was carried out at a constant potential of 80 mV (vs. RHE) for 20 h. The stability test was conducted by repeating the CV scan at a rate of 100 mV/s for more than 10000 cycles from 0 to 0.3V (vs. RHE). Tafel slopes were determined by fitting the linear portion of the curve starting from the onset potential to a straight line based on the equation (S1):

$$\eta = b \log j + c \qquad \text{(S1)}$$

where b is the Tafel slope and j is the current density.

The exchange current density (j_0) was determined from the following equation (S2):

$$J_0 = e^{(-2.303c/b)} \qquad \text{(S2)}$$

where b and c are the Tafel slope and intercept, respectively, in equation (S1).

Supplementary Note 5. Computational Details. Spin-polarized density functional theory (DFT) computations were performed using the Vienna Ab-initio Simulation Package (VASP) with the projector-augmented-wave (PAW) method to account for core-valence interactions[10-12]. The exchange-correlation interactions were described by generalized gradient approximation (GGA) with Perdew-Burke-Ernzerhof (PBE) functional[13]. Moreover, van der Waals correction was also

incorporated using an empirical scheme by Grimme as implemented within the framework of VASP for DFT-D3 method[14]. A polarized continuum model of Hennig and co-workers implemented in VAPS as VASPsol was used to simulate the effect of a water solvent environment with the dielectric constant set to 78.4[15]. For a modeling of 2D surfaces, we considered (3x3) and (4x2) supercells for 2H and T′ phases of MX_2, respectively, with a vacuum of 20 Å along *z*-direction to avoid undesired interactions due to the periodic images (Supplementary Fig. 15A,B). A cutoff energy of 500 eV was used to generate plane wave basis set and the Brillouin zone was sampled using 3x3x1 Monkhorst-Pack k-points mesh[16]. All the structures were relaxed until the convergence criterion of 10^{-5} eV and 0.05 eV $Å^{-1}$ were met for self-consistent field (SCF) energies and forces on each atom, respectively. In order to test the

HER activity on the edge sites of 1T′ MX_2, we modeled a hydrogen passivated nanoribbon (Supplementary Fig. 15F) structure with a vacuum of 20 Å in the y-direction. Two kinds of edge structures are created by the above procedure, including the one that ends with the exposed metal atoms (Mo/W) and the other ending with chalcogen only (S/Se/Te).

All the dangling bonds on these edges are capped by the H atoms.

We considered efficient Volmer-Heyrovsky reaction mechanism for the evaluation of theoretical overpotential for HER at the surface and edges of MX_2 systems[17].

$$* + (H^+ + e^-) \rightarrow *H \qquad \text{(Volmer step)} \qquad \text{(S3)}$$

$$*H + (H^+ + e^-) \rightarrow * + H_2 \qquad \text{(Heyrovsky step)} \qquad \text{(S4)}$$

Gibbs free energies of binding for the reaction steps were calculated while considering the Norskov′s computational hydrogen electrode model, which approximates the chemical potential of a proton–electron pair to be equivalent to that of half of the hydrogen gas at standard conditions ($\mu(H^+ + e^-) = 0.5\mu(H_2)$ at pH= 0, 101 325 Pa of H_2, 298.15 K)[18-20]. The effect from electrode potential on the chemical potential of an electron could be expressed as $\Delta G_U = -neU$, where n is

the number of electrons transferred and U is the applied electrode potential. The explicit contribution to ΔG is defined as:

$$\Delta G(*H) = \Delta E_{DFT}(*H) + \Delta E_{zpe} + T\Delta S + \Delta G_U \quad (S5)$$

where the reaction energy ΔE_{DFT} is directly obtained from DFT calculations, ΔE_{zpe} is the change in zero-point energy, T is the temperature (298.15 K), and ΔS is the change in entropy[21]. Contribution of free energy from pH is assumed to be zero for an acidic medium. The entropies and the vibrational frequencies of adsorbed H and H_2 molecules in the gas-phase were calculated using the harmonic oscillator approximation at 298.15 K. The ZPE values for H_2 molecule, *H (T′ MX_2) and *H (T′ MX_2 NR) are calculated as 0.27 eV, 0.23 eV and 0.18 eV, respectively. However, TS value for H_2 molecule is 0.41 eV and approximately zero for all the H adsorbed systems. Changes in the free energies given in Fig. 5A (main text) is that of the exposed metal edge sites, which are the most active sites for HER in the MX_2 NR structures.

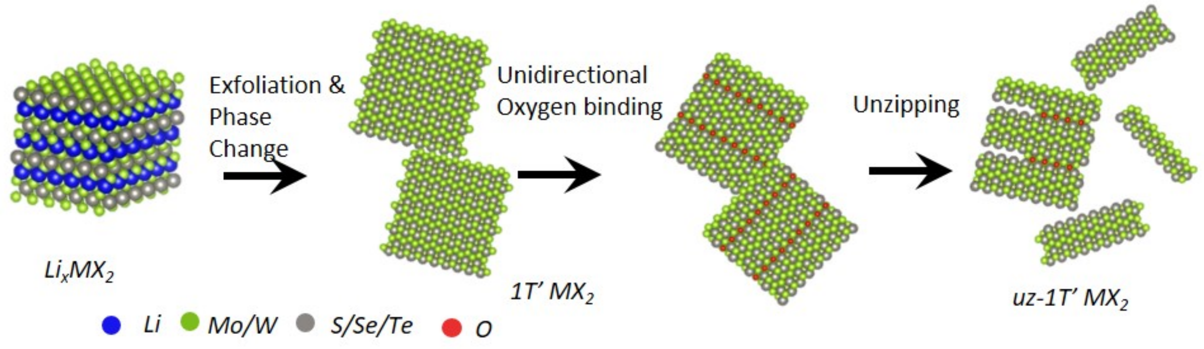

Supplementary Fig. 1. Unzipping MX_2. Schematic illustration of unzipping procedure from bulk MX_2.

Supplementary Note 6. XPS and Raman spectroscopy characterization of WSe_2 NRs. High resolution W4f -XPS spectra of WSe_2 NRs compared to precursor (bulk WSe_2 flakes) and randomly etched WSe_2 sheets are given in Supplementary Fig. 2D. W4f binding energies of pure 2H phase bulk WSe_2 flakes are located at ~34.58 ($4f_{5/2}$) and 32.40 ($4f_{7/2}$) eV. In the XPS spectra of WSe_2 NRs, both the $4f_{5/2}$ and $4f_{7/2}$ peaks have shifted to lower (~0.9 eV) binding energies assigned to 1T′ phase. The lower binding energy of W4f peaks in 1T′ phase from those of 2H phase is due to the change in metal coordination and W-Se bond length associated with the phase change[6]. The mixture phase (1T′ and 2H) WSe_2 sheets exfoliated in oxygenated water show the predominant oxidation peaks at ~35.74 and ~37.92 eV compared to NRs. Raman spectrum (Supplementary Fig. 2E) of bulk WSe_2 displayed two unresolved high intensity peaks corresponding to in-plane E^1_{2g} (248.8 cm^{-1}) and out-of-plane A_{1g} (258.1 cm^{-1}) vibration modes of pure 2H phase. By contrast, Raman spectrum of WSe_2 NRs shows many vibrational modes with lower intensities, typical of 1T′ metallic phase. Similar to MoS_2 NRs, the peak for in-plane vibration mode (E^1_{2g}) is absent in the spectrum of WSe_2 NRs, but a small intensity out-of-plane vibration mode (A_{1g}) can be observed, which can be associated to the high density of edge sites created upon the unzipping of the basal plane[22].

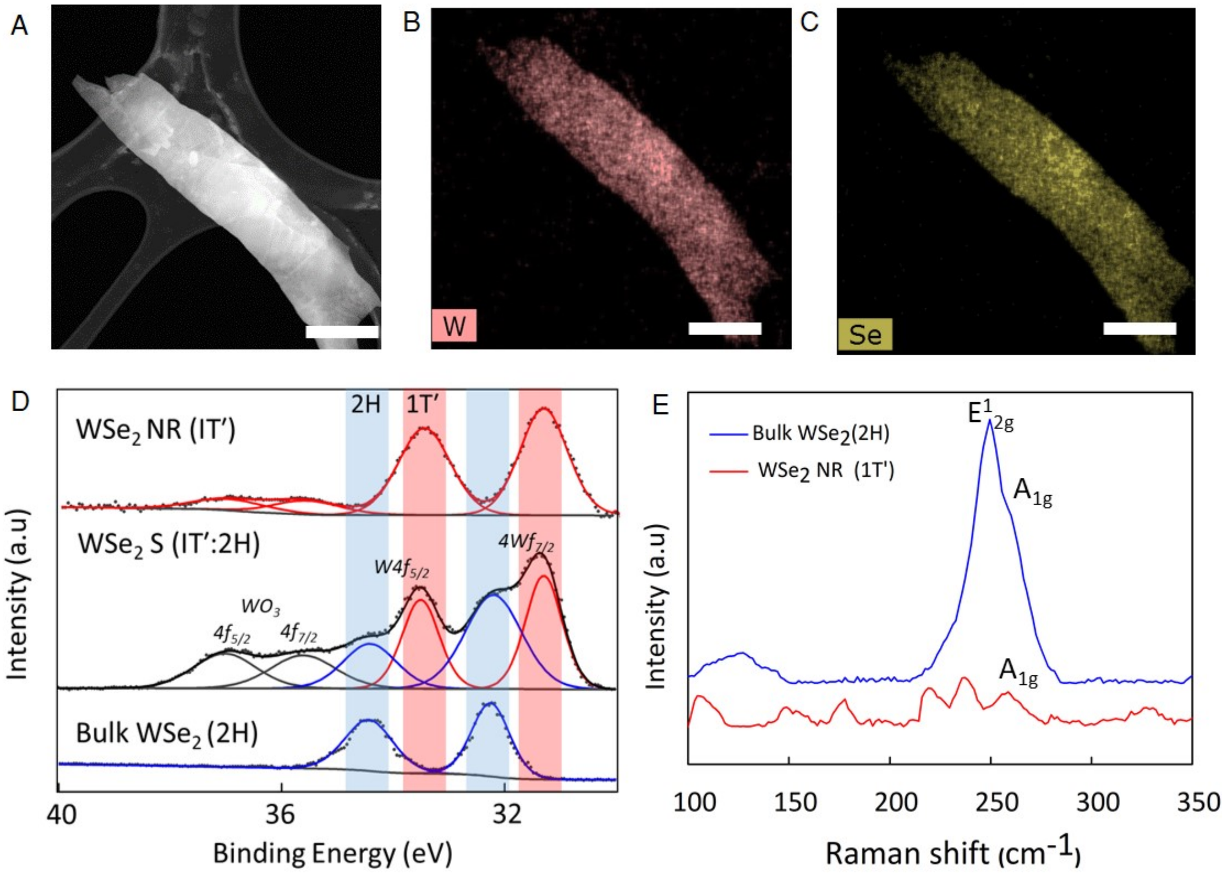

Supplementary Fig. 2. WSe_2 NRs. (A) High-angle annular dark-field scanning transmission electron microscopy (HAADF-STEM) and the associated elemental (B) W and (C) Se scanning results for WSe_2 nanoribbon. Scale bars, 0.1 μm. (D) XPS spectra of precursor bulk 2H WSe_2 flakes, WSe_2 NR and randomly etched WSe_2 sheets of combined 1T′ and 2H phase. (E) Raman spectra of WSe_2 NR compared to the precursor WSe_2 flakes.

Supplementary Note 7. XPS and Raman spectroscopy characterization of $MoSe_2$ NRs. Mo3d binding energies of pure 2H phase $MoSe_2$ (both bulk and 2D forms) are observed at ~232.1 ($3d_{3/2}$) and 229.0 ($3d_{5/2}$) eV in the high resolution XPS spectra (Supplementary Fig. 3D). In the XPS spectra of $MoSe_2$ NRs obtained by the unzipping of basal plane of 2D $MoSe_2$, the $3d_{3/2}$ and $3d_{5/2}$ peaks have shifted to lower binding energies of 231.2 and 228.1 eV, respectively, due to the different relaxation energies associated with 1T′ phase[23]. In the XPS spectrum of mixture phase (2H + 1T′) $MoSe_2$ sheets, all $3d_{3/2}$ and $3d_{5/2}$ peaks corresponding to 2H and 1T′ phase coexist. The mixture phase $MoSe_2$ tends to favour random oxygenation resulting in the etching of the basal plane to form porous $MoSe_2$ structures. Therefore, the peak at higher binding energy (>235 eV) arising from the presence of MoO_3 species is found to significantly higher in the mixture phase $MoSe_2$ sheets compared to $MoSe_2$ NRs, despite the same experimental procedure used for the unzipping basal plane of 1T′ $MoSe_2$ sheets. Raman spectrum (Supplementary Fig. 3E) of $MoSe_2$ flakes displays the characteristic in-plane E^1_g (165.1 cm^{-1}) and out-of-plane A_{1g} (236.3 cm^{-1}) vibration modes assigned to pure 2H phase. The Raman spectrum of $MoSe_2$ NRs shows many unresolved vibrational peaks (106.4, 150.8, and 221.3 cm^{-1} correspondings to J_1, J_2, and J_3 respectively) of weak intensities, indicating metallic phase[24]. The presence of A1g peak at 236.3 $cm^{-1}$and the absence of E^1_{2g} peak at 165.1 cm^{-1}corresponding to loss of in-plane vibration mode is due to the basal plane unzipping.

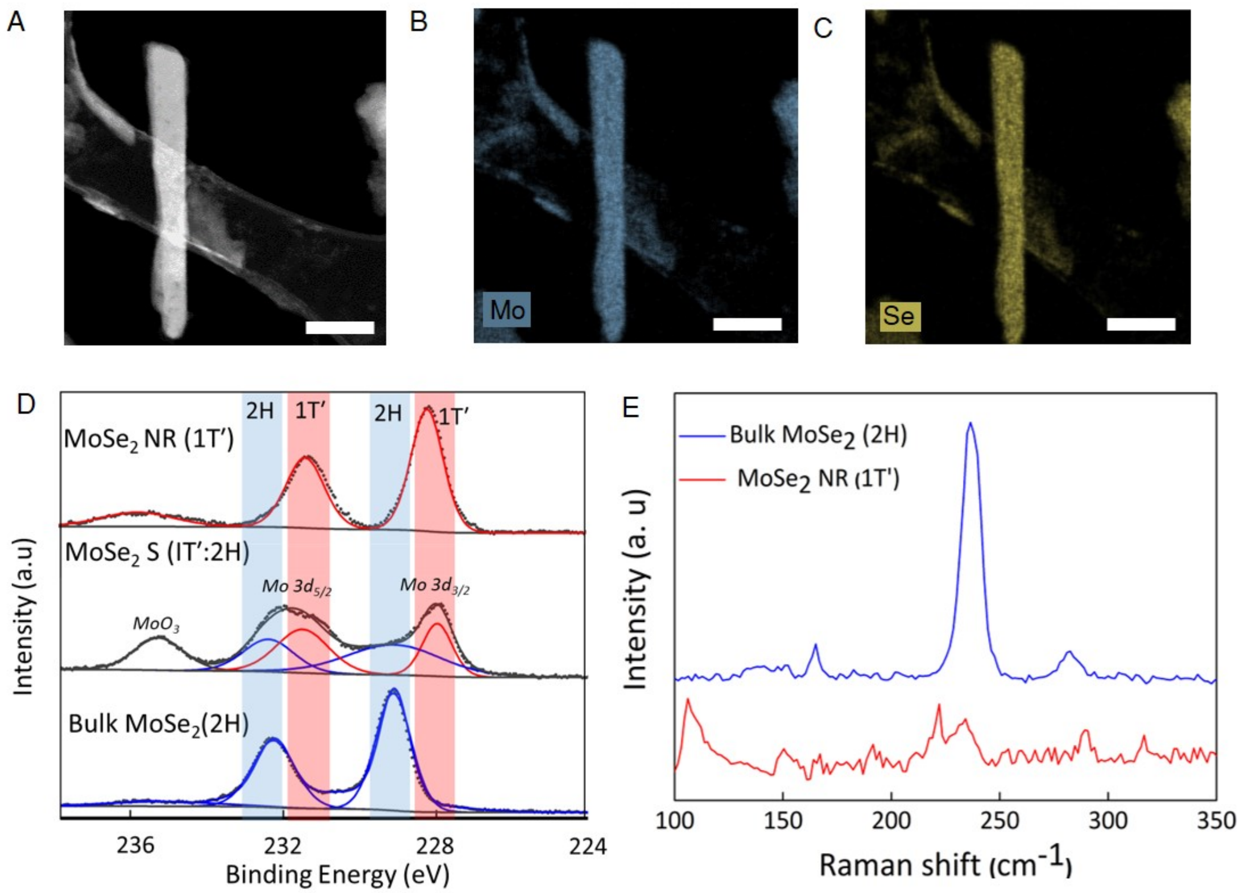

Supplementary Fig. 3. $MoSe_2$ NRs. (A) HAADF-STEM and the associated elemental (B) Mo and (C) Se scanning results for $MoSe_2$ NR. Scale bars, 0.2 µm. (D) XPS spectra of the precursor 2H $MoSe_2$ flakes, $MoSe_2$ NRs and randomly etched $MoSe_2$ sheets of combined 1T′ and 2H phase. (E) Raman spectra of $MoSe_2$ NRs compared to the precursor $MoSe_2$ flakes.

Supplementary Note 8. XPS and Raman spectroscopy characterization of $MoTe_2$ NRs. Similar to the XPS spectra of MoS_2 and $MoSe_2$ NRs, the Mo3d binding energies of 2H phase $MoTe_2$ (227.8 and 230.9 eV) have been downshifted by ~0.9 eV in the spectrum of $MoTe_2$ NRs (Supplementary Fig. 4D). Raman spectrum (Supplementary Fig. 4E) of $MoTe_2$ flakes displayed two high intensity peaks at 172 and 233 cm^{-1} assigned to out-of-plane A^1_g and in-plane E^1_{2g} vibrational modes of pure 2H phase[25]. The Raman spectrum of $MoTe_2$ NRs show three low intensity vibrational peaks at 83.3, 164.7, and 269.4 cm^{-1} correspondings to 1T′ $MoTe_2$[26].

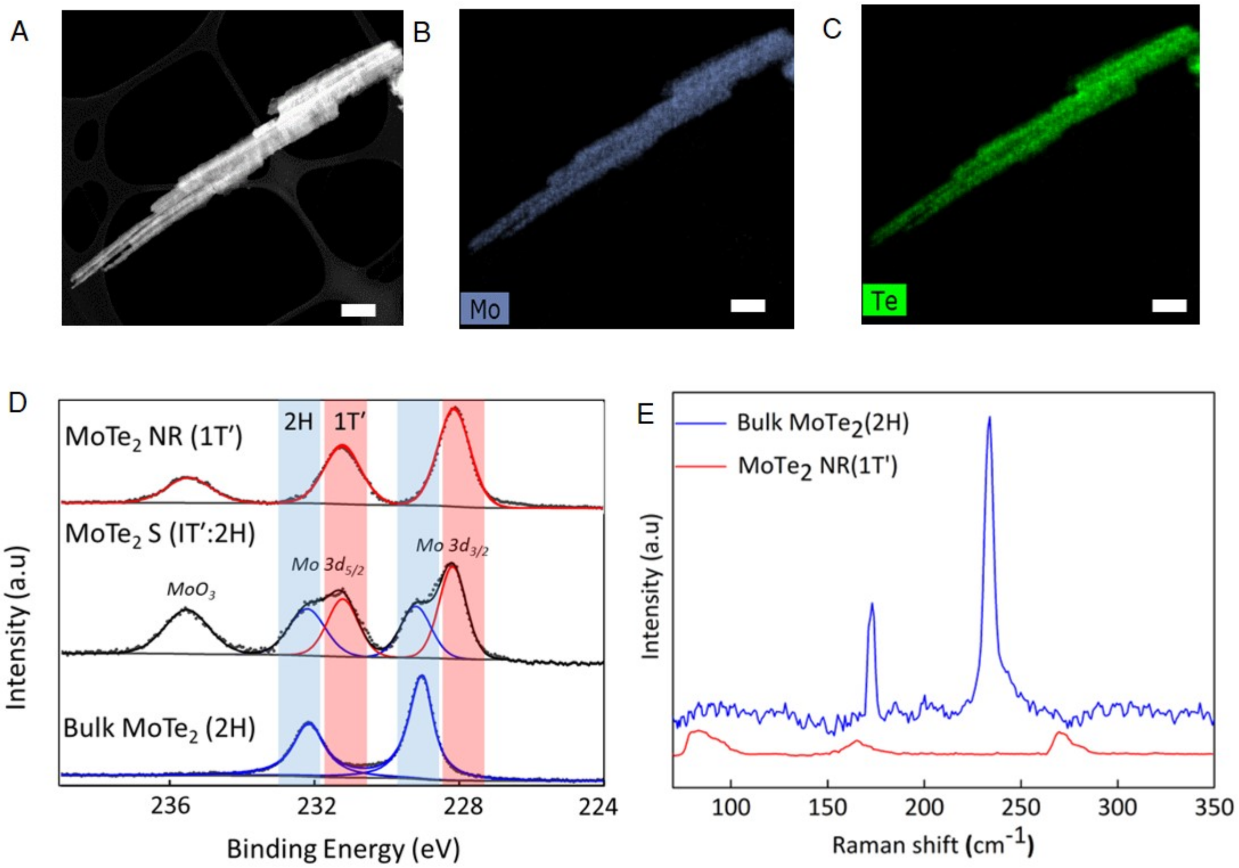

Supplementary Fig. 4. $MoTe_2$ NRs. (A) HAADF-STEM and the associated elemental (B) Mo and (C) Te scanning results for $MoTe_2$ NR. Scale bars, 0.1 µm. (D) XPS spectra of the precursor 2H $MoTe_2$ flakes, $MoTe_2$ NRs and randomly etched $MoTe_2$ sheets of combined 1T′ and 2H phase. (E) Raman spectra of 1T′ $MoTe_2$ NRs compared to the precursor $MoTe_2$ flakes.

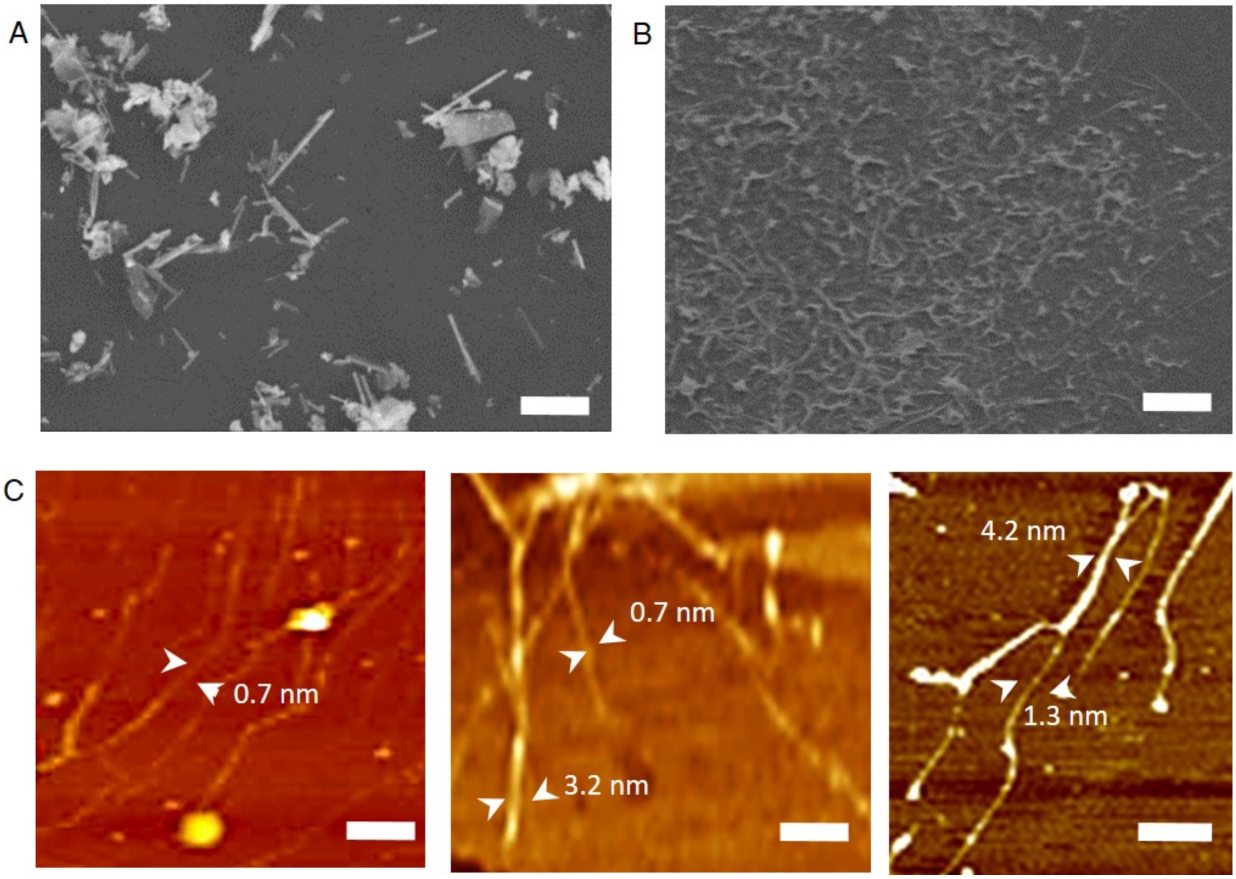

Supplementary Fig. 5. MoS_2 NRs. (A) SEM image of unzipped MoS_2 after ultrasonication displaying the different structures, including nanoribbons, nanosheets and bulk nanoparticles (B) SEM image of the concentrated MoS_2 NRs after the sequential centrifugation and removal of thick layered structures and debris. SEM images are obtained by tilting the sample holder to 60-90° to increase the sample contrast. Scale bars, (A) 1 and (B) 0.3 µm. (C) Representative AFM images of MoS_2NRs displaying the thicknesses of 0.7 to 4.2 nm, corresponding to 1 to 5 layers, respectively. Scale bars, 0.5 µm.

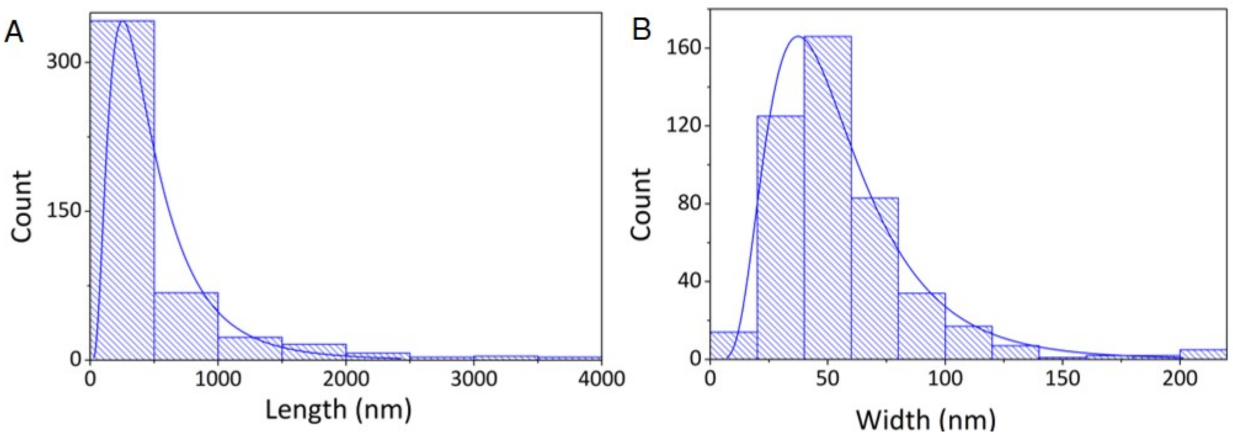

Supplementary Fig. 6. Dimensions of MoS_2 NRs. Histograms showing average (A) length and (B) width distributions for MoS_2 NRs. More than 400 fully unzipped MoS_2 NRs were considered for the statistical results.

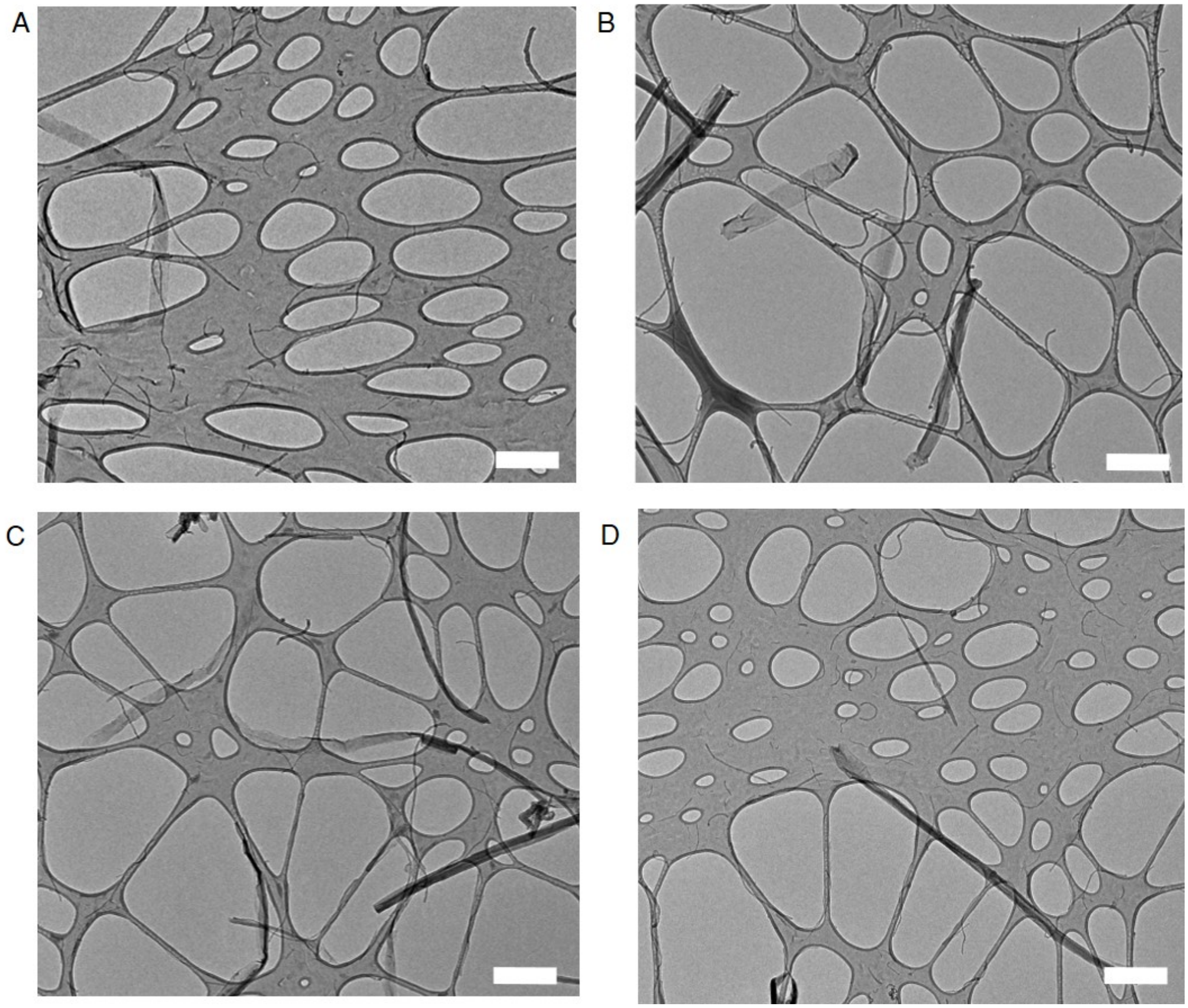

Supplementary Fig. 7. Large area TEM of MX_2 NRs displaying a wide size distribution. Scale bars, 0.5 µm.

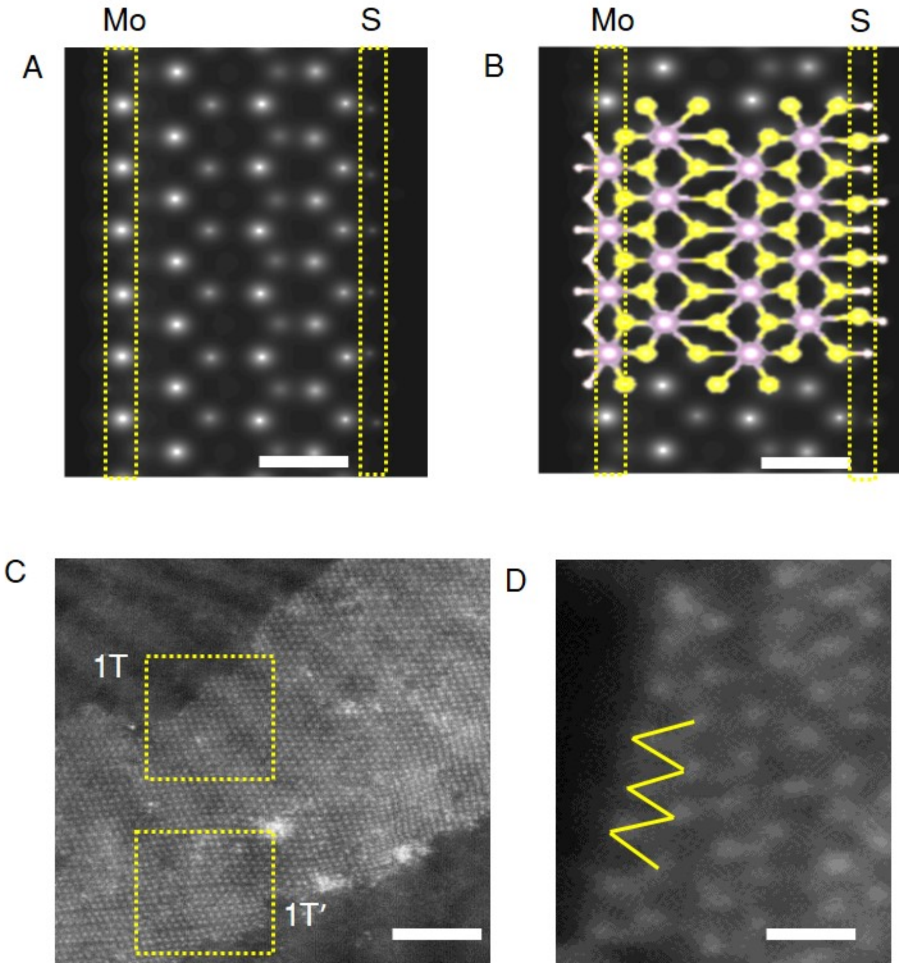

Supplementary Fig. 8. Edge structure of MoS_2NR. (A) Simulated STEM image with (B) the DFT optimized MoS_2NR atomic structure showing Mo terminating zig-zag 1T′ edge on one side and S terminating zig-zag 1T′ edge on the other side. Edges are passivated by hydrogen in the DFT optimized MoS_2NR atomic structure for stability. (C) Experimentally obtained STEM image of MoS_2NR. (D) Magnified edge structure displaying Mo terminated zig-zag 1T′ edges. Scale bars, (A, B) 0.2 (C) 4 and (D) 0.25 nm. All the edge sites are Mo terminated in the STEM image provided in (C), albeit displays both 1T and 1T′ structures (due to the low stability of 1T′ phase under electron beam).

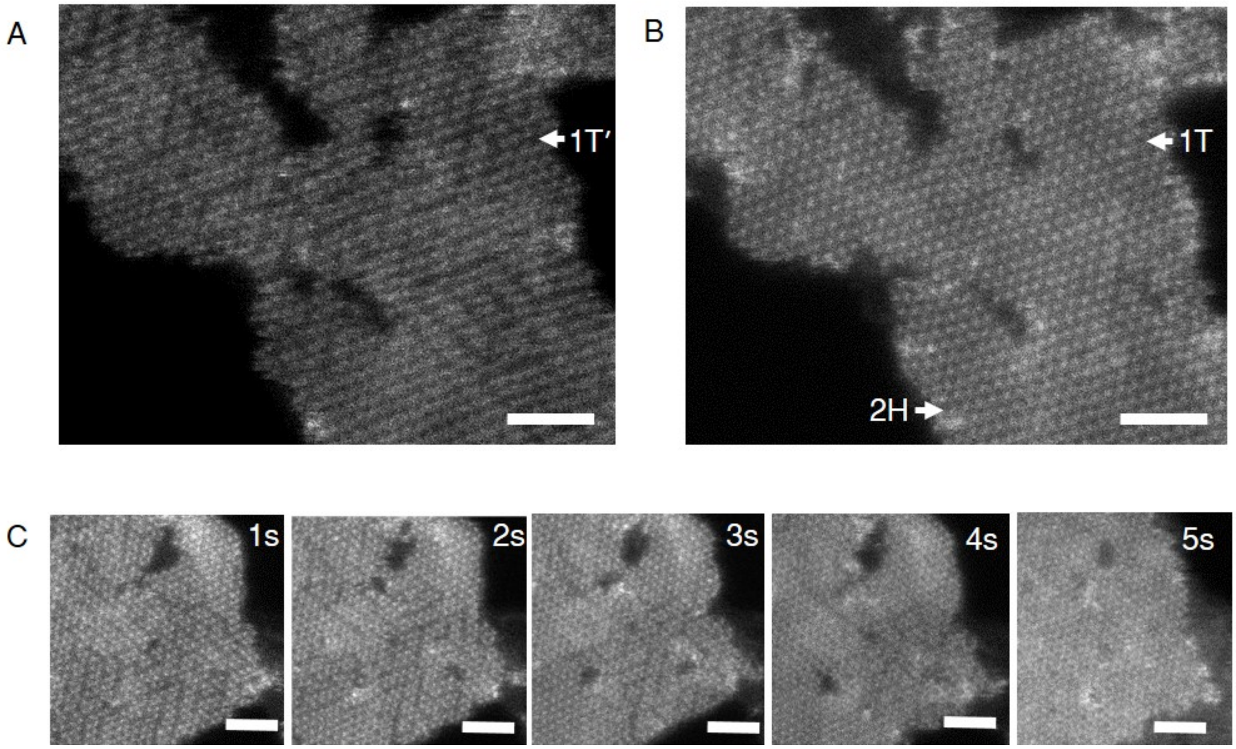

Supplementary Fig. 9. Stability of 1T′ phase under electron beam exposure. (A) HAADF STEM image of MoS_2 NR showing 1T′ structure, (B) which converts to mixed 1T and 2H phase after 5s of electron beam exposure. (C) HAADF STEM images of 1T′ MoS_2 under continuous e-beam exposure up to 5s displaying gradual phase change. Scale bars, (A-C) 2 nm. As shown, all the edge sites are Mo terminated.

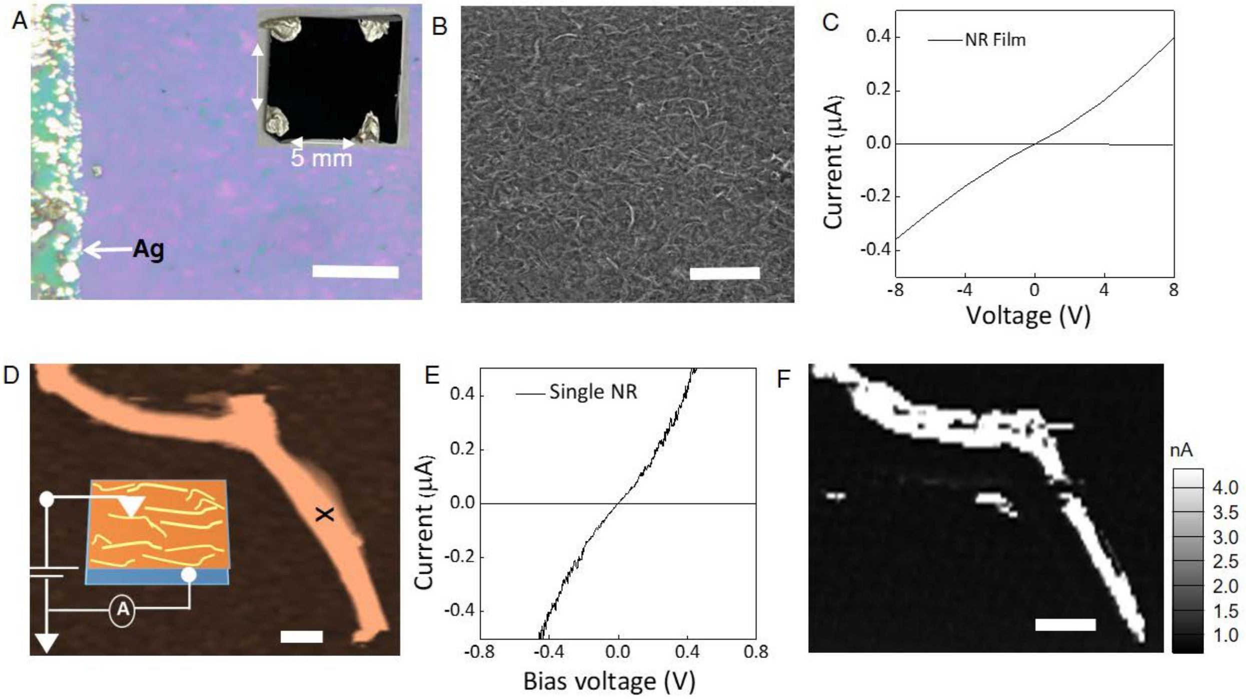

Supplementary Fig. 10. Electrical characteristics of MoS_2 NRs. (A) Optical image and (B) SEM of thin film of MoS_2 NRs on Si/SiO_2 surface used for electrical measurement. The silver contact point is also included in the optical image. Scale bars, (A) 30 µm and (B) 1µm. Photograph of tested sample is provided in the inset of A. (C) I-V curve of MoS_2 NR film showing a typical Ohmic characteristic. (D) Conductive AFM topography image of single NR of thickness 3 nm and (E) I-V curve measured by AFM tip on the NR surface from the spot marked X. Measurement set-up is schematically presented in the inset of D. (F) Current map of single NR measured at an applied voltage of 200 mV revealing discrepancy in the current density. Scale bars (D)100 nm and (F) 200 nm.

Supplementary Note 9. Phase change of MoS_2 NRs by temperature. The extent of phase change at different temperatures has been evaluated by XPS (Supplementary Fig. 11A). The MoS_2 NRs showed the characteristic peaks at 227.9 ($Mo3d_{5/2}$), 231.1 ($Mo3d_{3/2}$) and 225.1 eV (S2s), representative of 1T′ MoS_2. The deconvolution of the $Mo3d_{5/2}$ and $Mo3d_{3/2}$ peaks showed two additional peaks at 228.8 and 232.1 eV, respectively, corresponding to 2H phase. The conversion ratio of 1T′ to 2H phase was calculated from the relative peak areas. The major transition occurred between 100 and 200 °C. Raman spectrum of the MoS_2NRs samples (Supplementary Fig. 11B) showed the signature peaks of 1T′ phase MoS_2 at 156 (J_1), 228 (J_2) and 330 cm^{-1} (J_3) together with the out-of-plane vibration modes at 405.5 eV (A_{1g}), which have quenched with increasing temperature above 100 °C.

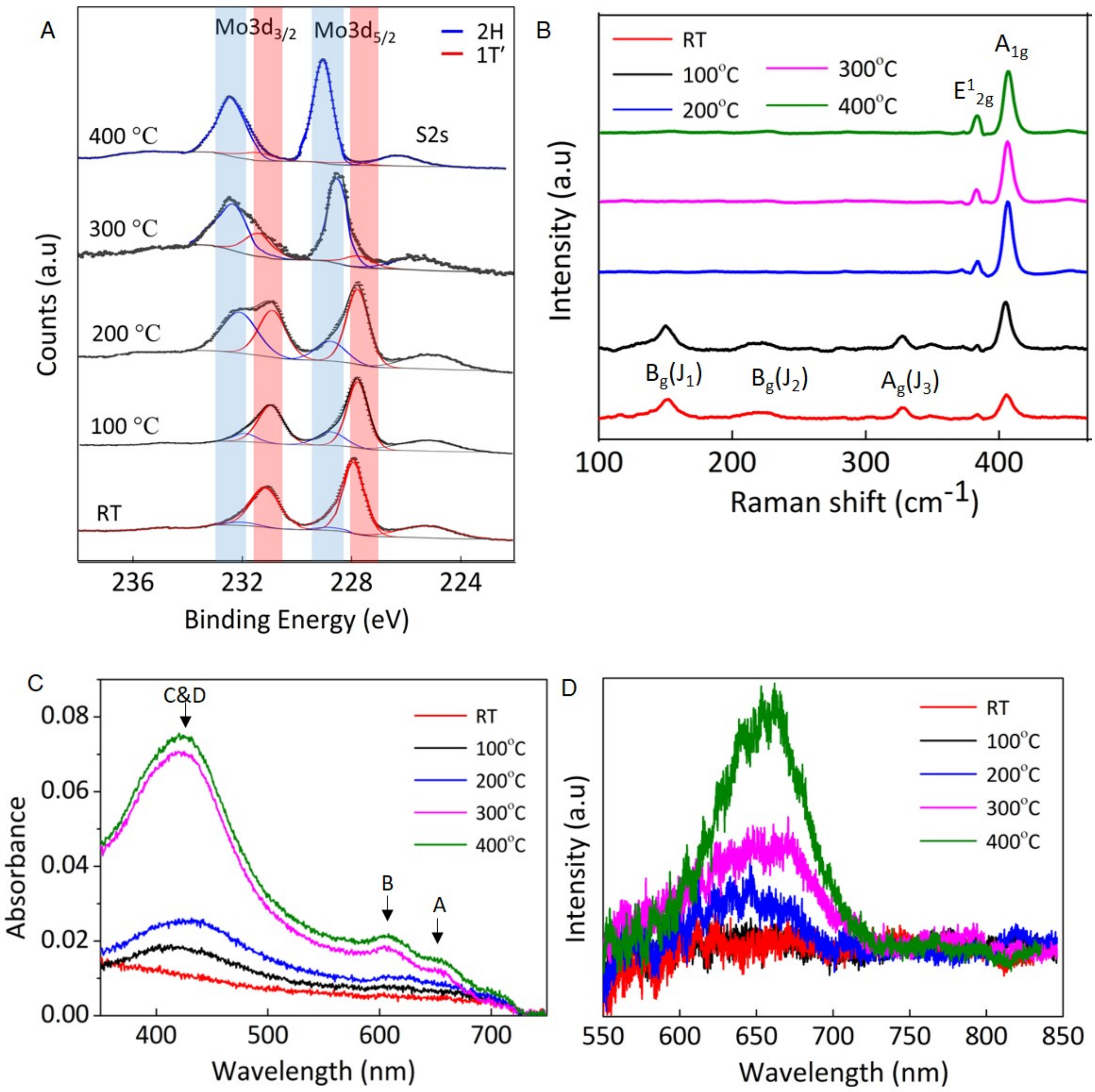

Supplementary Fig. 11. Effect of thermal annealing. (A) High-resolution XPS spectra of Mo3d and S2s peak regions. (B) Raman spectra, (C) UV-Visible spectra, and (D) PL spectra of MoS_2 NRs annealed at various temperatures.

Supplementary Note 10. Phase dependent oxidation in MoS_2. 1T′ MoS_2 sheets were prepared by the treatment of bulk MoS_2 (which have an intrinsic 2H phase) with 0.48 M *n*-BuLi for 48 h at 90 °C (more details are given in supplementary note 1). The sample was then ultrasonicated for 15 min in the oxygenated water and centrifuged several times to recover partially unzipped MoS_2 in the supernatant. Similar procedure was followed for the mixed phase (2H: 1T′) MoS_2 with modified reaction parameters only for the *n*-BuLi intercalation step (0.48 M *n*-BuLi for 20 h at 60 °C). Other conditions including the 15 min of ultrasonication time was same. 2H MoS_2 sheets were obtained from the liquid phase exfoliation. As shown in the TEM images (Supplementary Fig. 12A, C and E), morphological evolution is strikingly different under the similar oxidation condition of MoS_2 depending on the initial crystalline phase. While partially unzipped MoS_2 sheet with exposed ribbon morphology from the edge of pure 1T′ phase was obtained, randomly etched porous sheet structures were obtained for both mixed phase and 2H phase after ultrasonication in oxygenated water. The resultant crystalline phase was confirmed by STEM images (Supplementary Fig. 12B, D and F). XPS spectroscopy (Fig. 3C in main text) confirms the high oxygen content in the randomly etched porous sheets of both mixed phase and 2H phase. XRD patterns of the exfoliated MoS_2 (MoS_2 S) with 2H and mixed-phase and partially unzipped 1T' MoS_2 (uz-MoS_2) showed a single broad reflection at 6.4 Å (Supplementary Fig. 12G,H). By contrast, bulk MoS_2 displayed a sharp and high intensity (002) peak at 6.2 Å. Such modified reflections for MoS_2S and uz-MoS_2 indicate the largely diminished layered structure along the *c* axis compared to bulk MoS_2. Interestingly, no peak representing the oxidized Molybdenum (MO_x) is present in the XRD patterns.

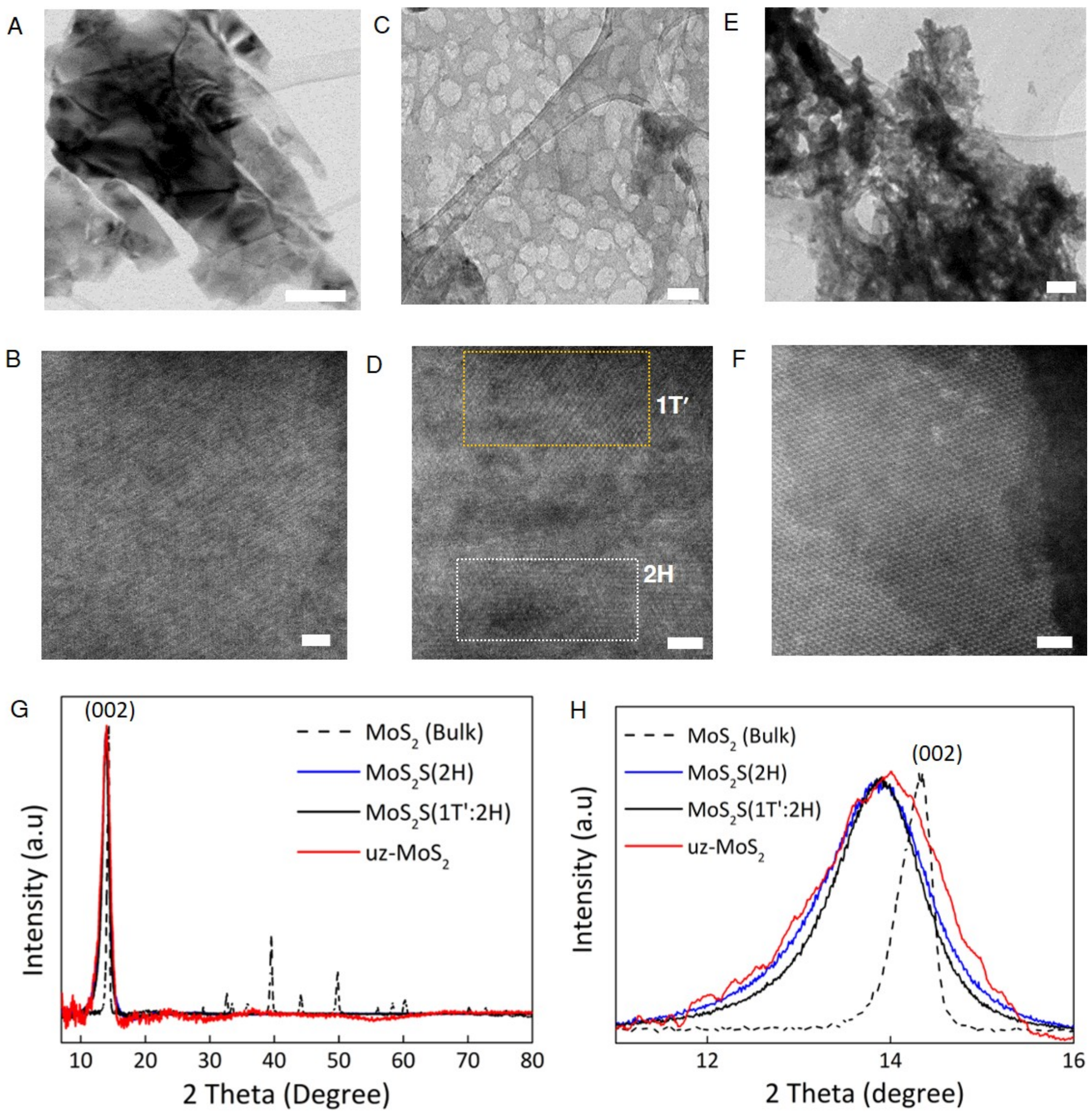

Supplementary Fig. 12. **Phase dependent oxidation behavior of MX2**. TEM (scale bar 100 nm) and STEM (scale bar 2 nm) images of (A, B) partially unzipped 1T′ MoS_2 sheet, and randomly etched MoS_2 sheets with (C, D) mixture phase (1T′ and 2H) phase and (E,F) pure 2H phase. (G) Intensity normalized XRD spectra compared to the precursor MoS_2 (Bulk), displaying only (002) peak in the partially unzipped and oxidized nanosheets. (H) Zoom in (002) peak region displaying the peak shift due to exfoliation.

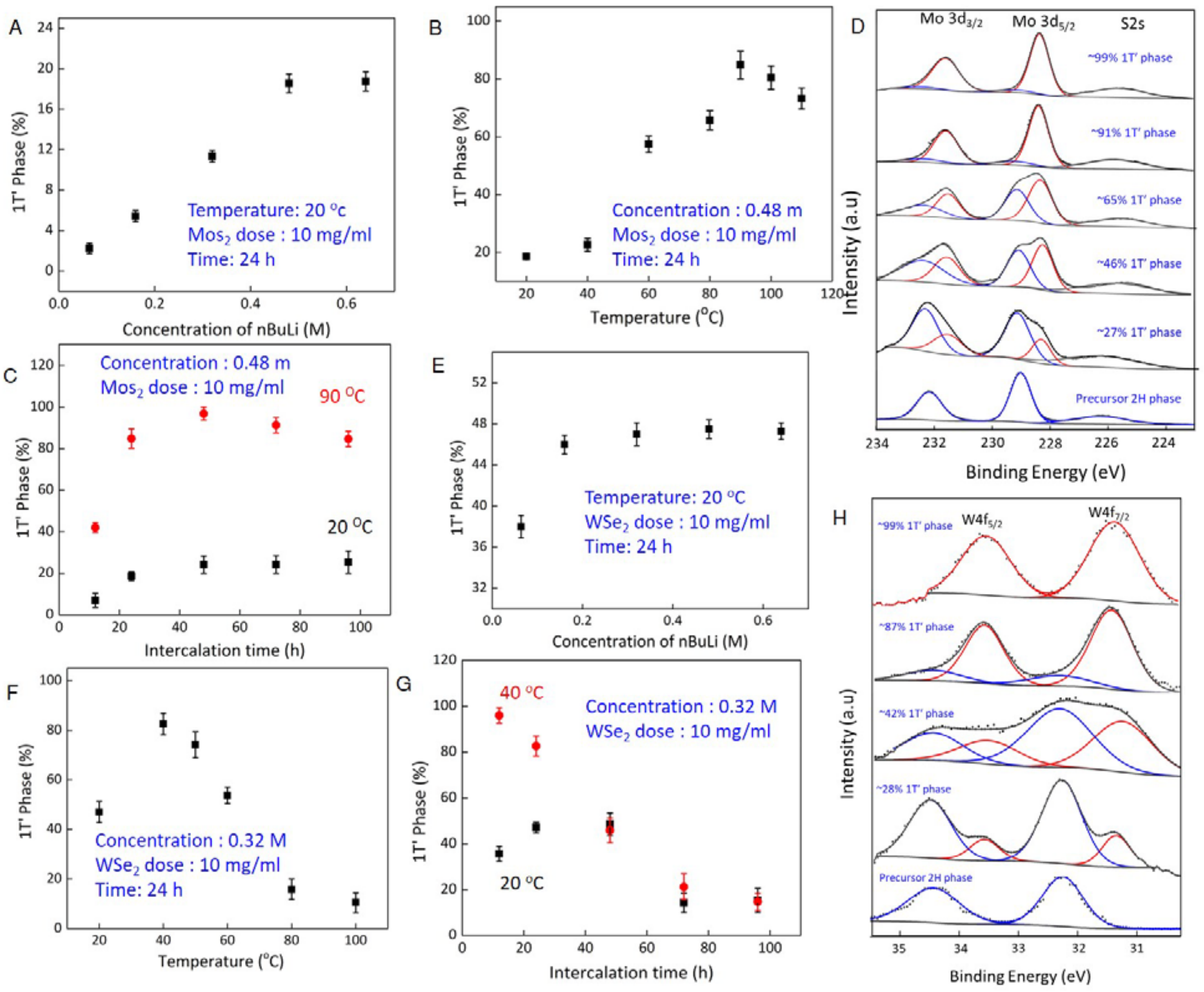

Supplementary Fig. 13. Evolution of 1T′ Phase in MoS_2 and WSe_2. Evolution of 1T′ Phase in exfoliated MoS_2 and WSe_2 sheets with respect to the (A,E) initial concentration of *n*-BuLi, (B,F) reaction temperature and (C,G) reaction time used to intercalate Li^+ into bulk crystals. (D, H) XPS spectra showing the evolution of 1T′ phase from pure 2H phase (precursor) for MoS_2 and WSe_2, respectively.

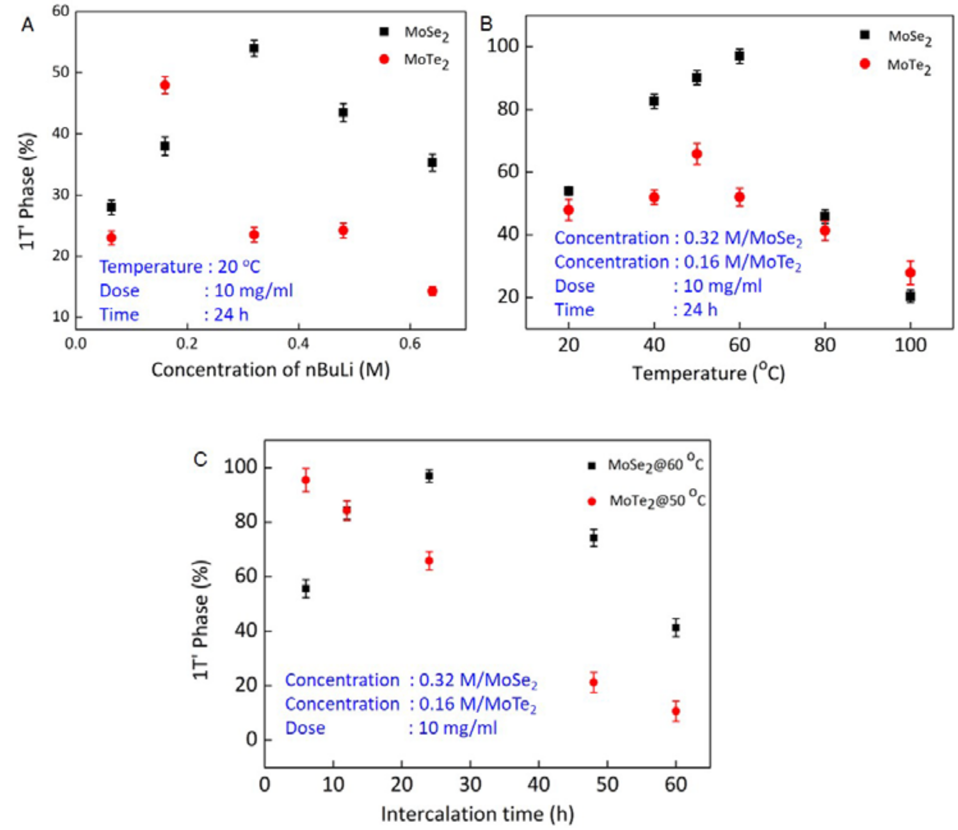

Supplementary Fig. 14. Evolution of 1T′ Phase in $MoSe_2$ and $MoTe_2$. Evolution of 1T′ Phase in the exfoliated $MoSe_2$ and $MoTe_2$ sheets with respect to (A) initial concentration of *n*-BuLi, (B) reaction temperature and (C) reaction time.

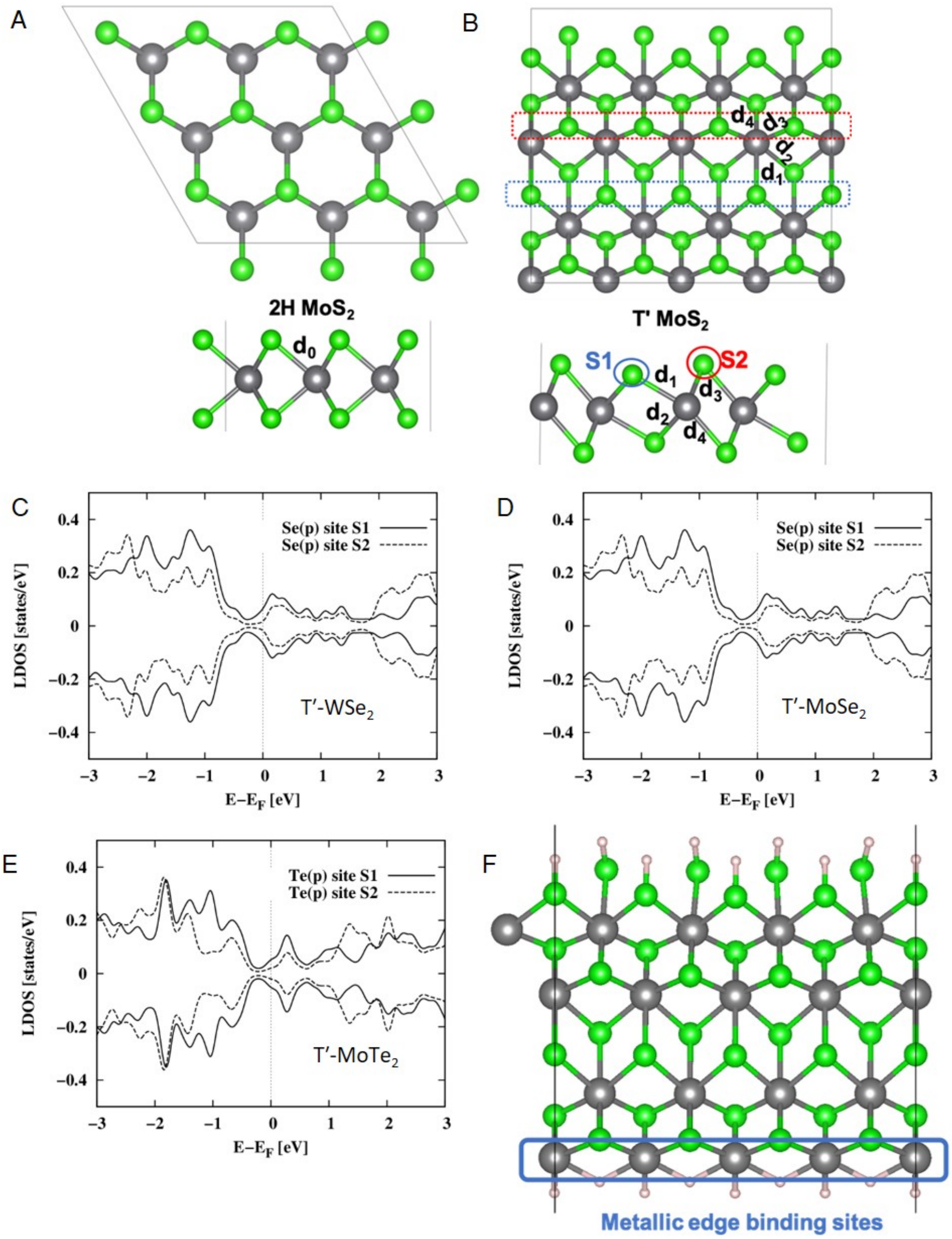

Supplementary Fig. 15. Active sites in 2H and 1T′ MX_2. Top and side views of considered supercells of (A) (3×3) 2H MoS_2, (B) (4×2) T′ MoS_2. Blue and red circles designate two possible S1 and S2 adsorption sites in 1T′, while all sites in 2H is same. Comparison of the local density of states (LDOS) corresponding to the chalcogen atoms at sites S1 and S2 in (C) 1T′ WSe_2, (D) 1T′ $MoSe_2$, and (E) 1T′ $MoTe_2$. The dashed lines correspond to the Fermi level rescaled to zero. (F) Top view of H passivated T′ MoS_2 NR displaying active sites on the edges of 1T′ MoS_2 NR supercell (12.61 Å × 11.40 Å). Calculated densities of active sites per unit length are given in

Supplementary Table 2 for all MX_2 systems; $MoSe_2$ (12.97 Å × 11.86 Å), $MoTe_2$ (13.54 Å × 12.72 Å, and WSe_2 (13.04 Å × 11.81 Å).

Supplementary Note 11. Local density of states (LDOS) analysis: LDOS diagram for X (S/Se/Te) atoms in 1T′ MX_2 (Fig. 2C and Supplementary Fig. 15C-E) show the existence of different degrees of 3p projected density of states for X atoms. The X atoms in S1 sites have a higher density of states at Fermi level, which reflects its strong binding nature towards O atom compared to the X atom at S2 site. This tendency has been consistent in all the tested MX_2 (MoS_2, $MoSe_2$, $MoTe_2$, and WSe_2).

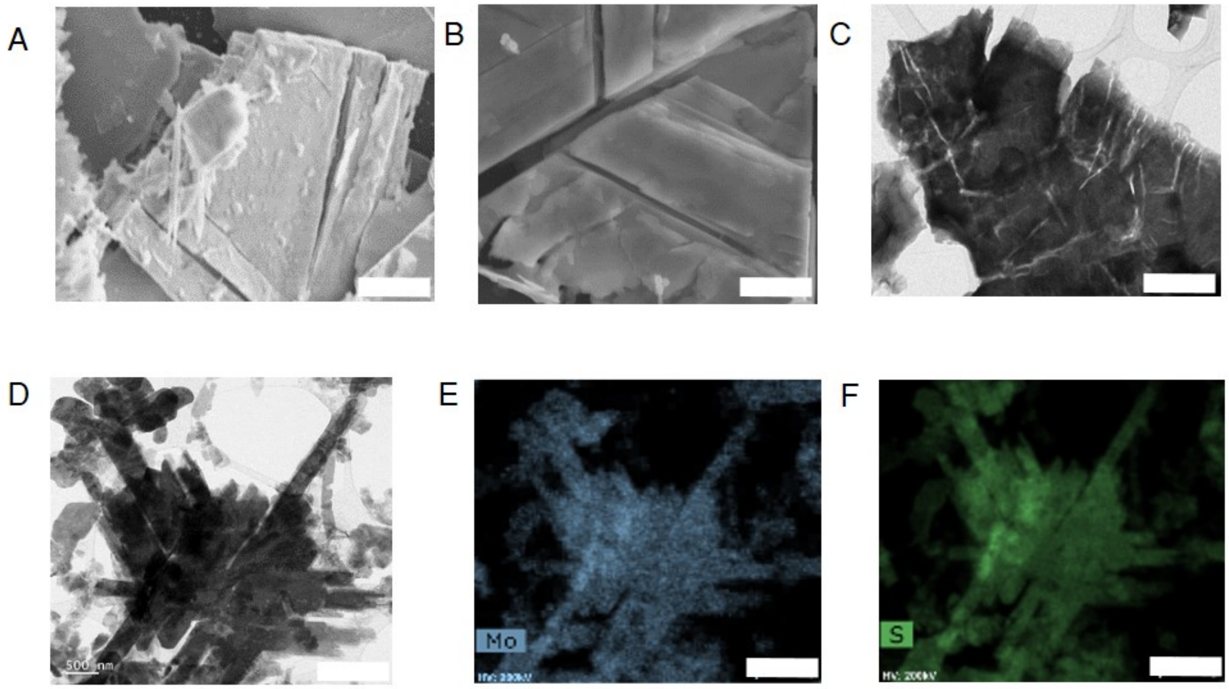

Supplementary Fig. 16. Evidence of line defects. (A-C) SEM images displaying line defects on the Li_xMoS_2 flakes upon hydration in oxygenated water. Scale bars, (A) 0.5 (B) 0.2 and (C) 0.1 µm. (D) TEM image and associated elemental (E) Mo and (F) S mapping results for partially unzipped MoS_2 after 30 min of ultrasonication in the oxygen-saturated water. Propagation of unzipping from the edge towards the center of a flake is clearly visible in all images. Scale bars, (D-F) 1 µm.

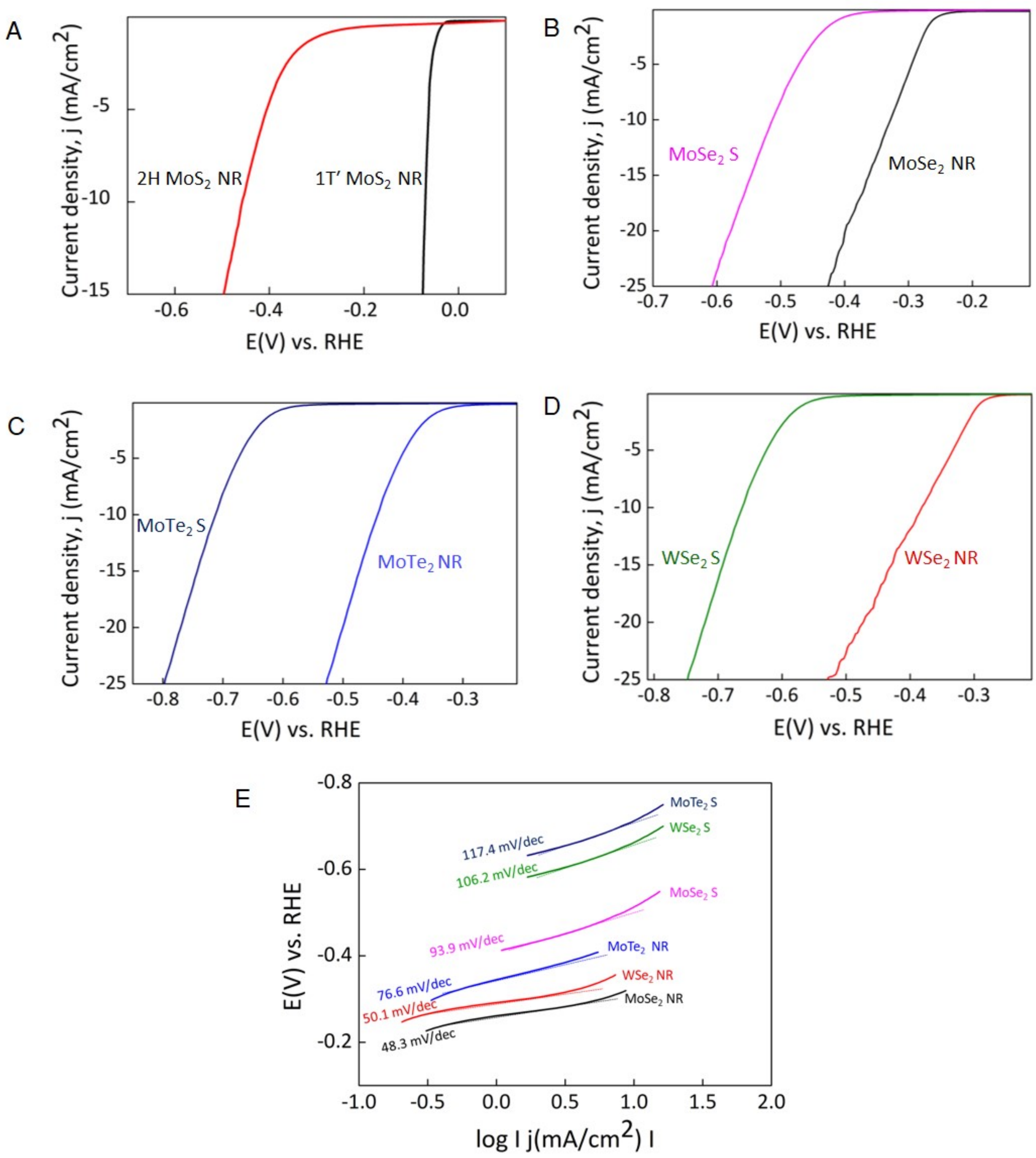

Supplementary Fig. 17. HER Catalytic activity and stability of MX_2 NRs. (A) Linear sweep voltammogram (polarization) curves of MoS_2 NRs before (1T′ phase) and after (2H phase) annealing at 400 °C. Polarization curves of (B) $MoSe_2$ (C) $MoTe_2$ and (D) WSe_2 NRs compared to corresponding 1T′ sheets and (D) corresponding Tafel plots. Dashed lines corresponds to linear fits considered for the calculation of Tafel slopes.

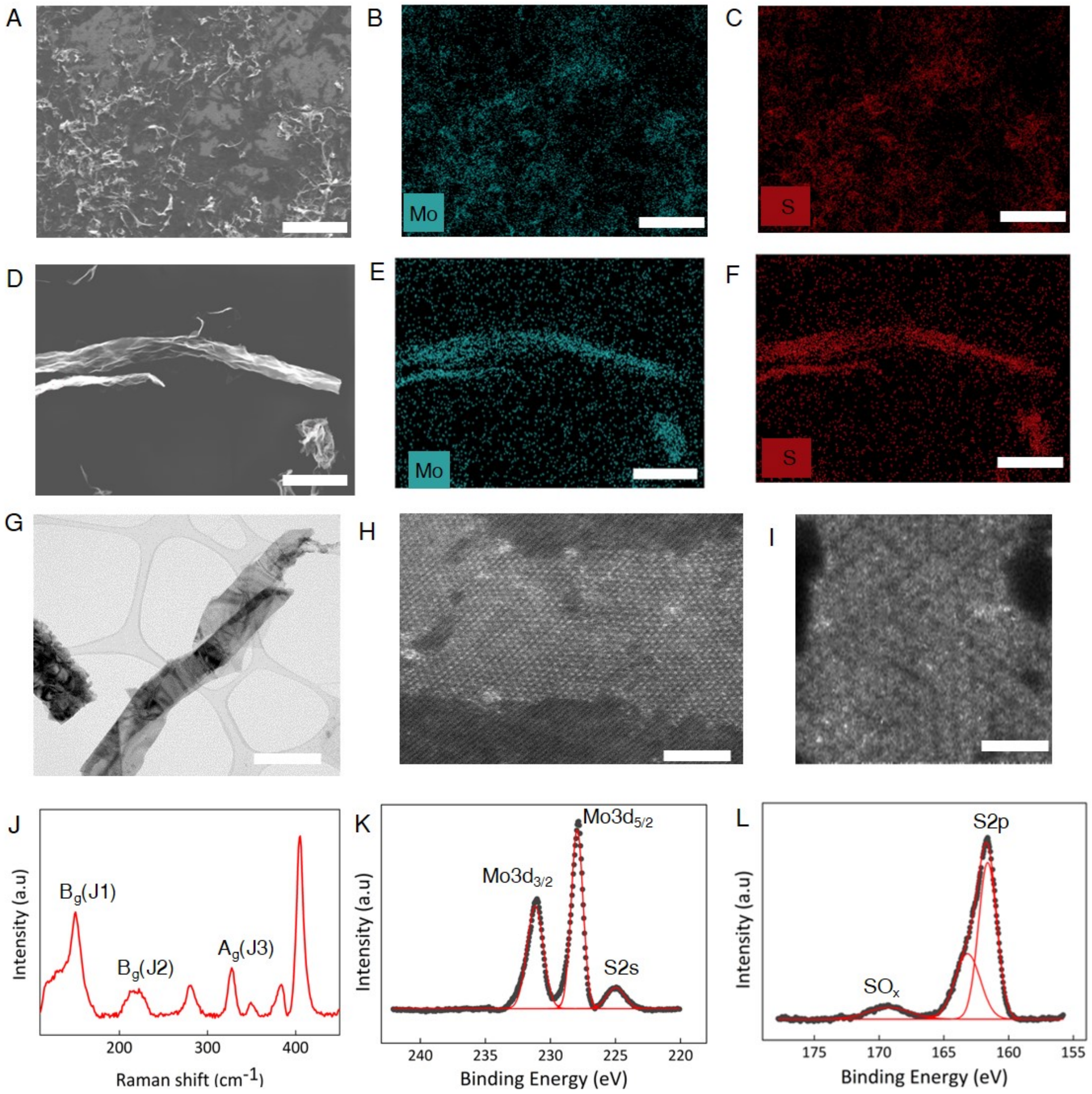

Supplementary Fig. 18. Morphological and chemical stability (A) SEM image of MoS_2 NRs after 10,000 CV cycles and the associated elemental (B) Mo and (C) S scanning results. (D) Zoom in SEM image of MoS_2 NR displaying wrinkled structure arising from amorphous contamination caused by nafion (incorporated for the electrode preparation), and associated elemental (E) Mo and (F) S scanning results. Samples were prepared by ultrasonication of the used electrode in ethanol for 3 min and directly dropping the obtained solution onto a clean Si/SiO_2 substrate. (G) TEM image of MoS_2 NRs. Samples were prepared by washing the used electrode materials with

ethanol by high speed centrifugation for a minimum of 3 times and drying in N_2 flow at 40 °C. (H, I) STEM images. The 1T′ structure is evident from STEM images. Scale bars, (G) 0.2 μm, (H) 3 and (I) 2 nm. (J) Raman and high resolution XPS spectra of (H) Mo_{3d} and (I) S_{2P} spectra of MoS_2 NRs displaying the excellent stability of 1T′ chemical structure. In the absence of MoO_3 peak (~235 eV), the presence of a small peak around 169 eV in S_{2P} spectra shows the oxidation of sulfur atoms.

Supplementary Note 12. Calculation of electrochemical surface area (ECSA) for MX_2 NRs.

The ECSA of the electrode was determined by measuring the electrochemical double layer capacitance (C_{dl}) obtained from the CV curves from 10 to 200 mV/s in the non-faradic region, as shown in supplementary Figure 19. The half of cathodic and anodic current density difference ($\Delta j/2$, $\Delta j=j_a-j_c$) is plotted against the scan rate. The absolute value of slope gives C_{dl}.

The ESCA is directly related to C_{dl} by the following equation [27]:

$$ESCA = C_{dl}/C_s \qquad (S6)$$

where C_s is the specific capacitance, which is generally considered to be in the range of 20-60 $\mu F/cm^2$ for a flat surface.

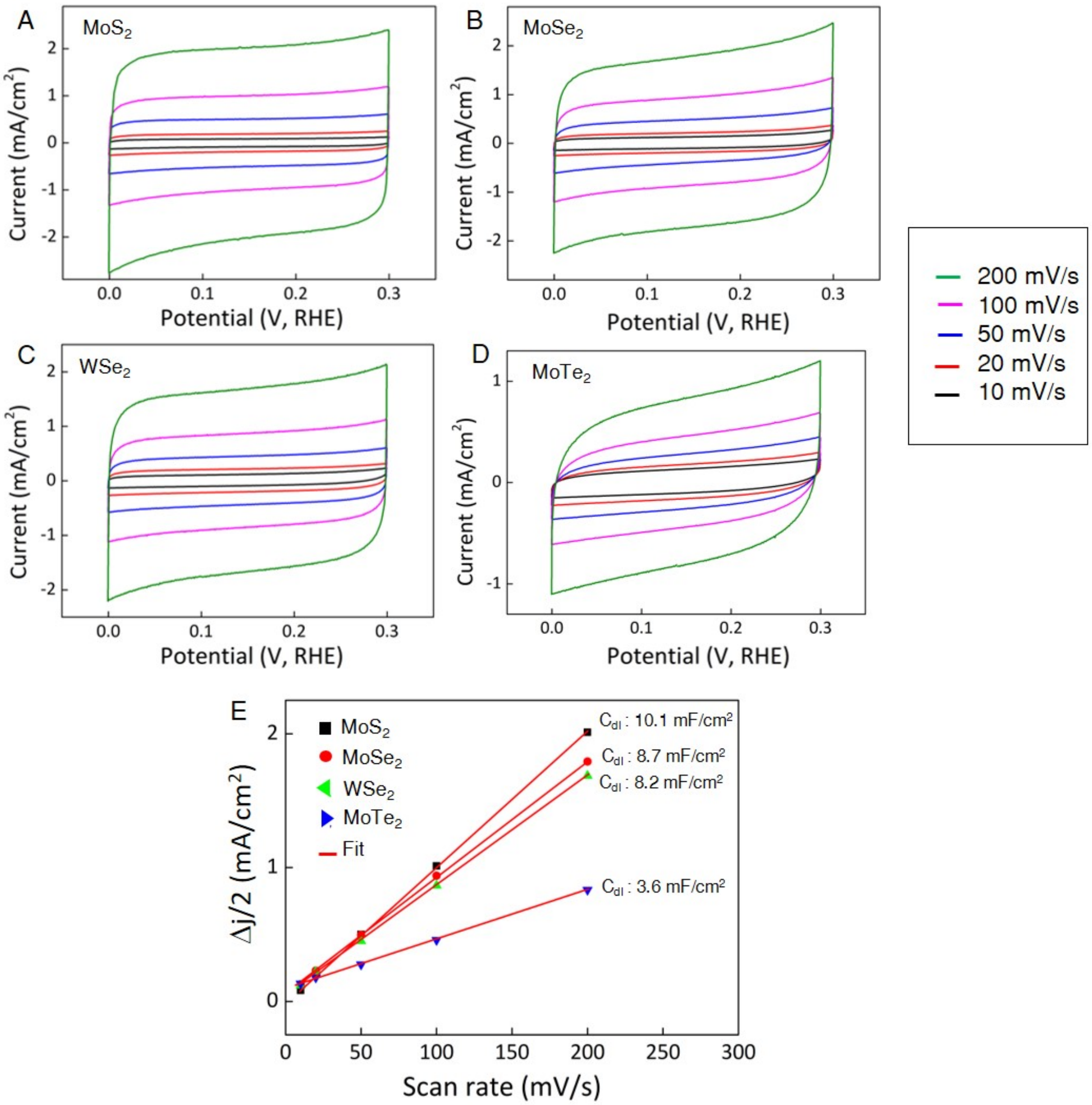

Supplementary Fig. 19. Double layer capacitance. (A-D) Electrochemical CV curves of different MX_2 NRs with increasing scan rates from 10 to 200 mV/s in the non-Faradic region. (E) The average of cathodic and anodic current density as a function of scan rate.

Supplementary Table 1. Comparison of HER activity of MX_2 NRs with the best characterized MX_2 materials in the literature.

MX_2 samples	Over potential (mV)	Tafel slope (mV/decade)	Exchange current density (A/cm^2)	TOF (s^{-1})	Double layer capacitance, C_{dl} (mF/cm^2)	Reference
1T′ MoS_2 (CVD)	110	55	$1.3x10^{-7}$	0.02	-------	28
Lithiated MoS_2/carbon fiber	113	43~47	$1.3\sim2.5x10^{-2}$	-------	-------	29
Epitaxial grown vertical $MoSe_2$<br>Epitaxial grown vertical MoS_2	~250<br>~250	105-120<br>105-120	$2x10^{-6}$<br>2.2×10^{-6}	0.014<br>0.013	-------<br>-------	30
2H phase<br>1T′ Phase (Chemical exfoliation)	>250<br>100	70-85<br>40	--------<br>--------	-------<br>-------	-------<br>-------	31
Oxygen incorporated MoS_2 (Hydrothermal)	300	55	$1.26\ x10^{-3}$	-------	0.037 mF	32
MoS_2 (CVD)<br>Oxygenated MoS_2	~200<br>~130	98<br>67	-------<br>-------	-------<br>-------	-------<br>-------	33
MoS_2 (Liquid phase exfoliation)	609	106	-------	-------	-------	34
1T′ MoS_2 (CVD+Li)	187	43	-------	-------	0.022 mF	35
1T′ WSe_2<br>1T′ MoS_2<br>1T′ $MoSe_2$	750<br>550<br>350	240<br>99<br>82	-------<br>-------<br>-------	-------<br>-------<br>-------	-------<br>-------<br>-------	7
Porous 1T′ MoS_2 (Hydrothermal synthesis+ chemical exfoliation)<br>Porous 2H phase MoS_2 (Hydrothermal)	156<br>274	42.7<br>75.3	-------<br>-------	-------<br>-------	14.8 mF<br>10.6 mF	36
1T′ MoS_2 (hydrothermal)<br>2H MoS_2	175<br>274	41<br>135	$1x10^{-4}$<br>$4x10^{-5}$	-------<br>-------	-------<br>-------	37
Porous 1T′ MoS_2 (Li+NH_3 liquid treatment)<br>Porous 2H MoS_2 (thermal treatment)<br>Porous 2H MoS_2 (Sulphur treatment)<br>1T′ MoS_2<br>2H MoS_2	153<br>218<br>257<br>203<br>343	43<br>62<br>82<br>48<br>106	$1.58x10^{-5}$<br>$1.05x10^{-5}$<br>$7.9x10^{-6}$<br>$1.26x10^{-5}$<br>$3.2x10^{-6}$	0.5<br>-------<br>-------<br>-------<br>-------	63.1<br>8.2<br>5.9<br>23.8<br>0.3	38
1T′ $MoSe_2$<br>1T′ WSe_2	380<br>780	65<br>120	-------<br>-------	-------<br>-------	-------<br>-------	39
MoS_2 defect rich (hydrothermal)	120	50	$8.9x10^{-6}$	0.725	-------	40
1T′ MoS_2 edge (CVD+ Li treatment+ selected area lithography)	77±24<br><br>201±42	60±4<br><br>68±10	$3.8\pm1.6x10^{-11}$<br>(A/cm)<br>$1.6\pm1.2x10^{-11}$	3.8±1.6<br><br>1.6±1.2	-------	41

2H MoS_2 edge (CVD+ selected area lithography)			(A/cm)			
MoS_2/strained S vacancies	170	60	-------	0.31	-------	42
MoS_2 monolayer flakes/Au foil	~278	73-85	(4.4-24.5) x10^{-6}	-------	-------	43
Hierarchical MoS_2 nanosheets	167	70	36 x10^{-6}	0.41	57.2	44
$MoSe_2$	340	88	5 x10^{-6}	-------	0.66	45
1T′ $MoTe_2$ (activated)	178	116	3.5 x10^{-5}	0.123	3.17	46
2H $MoTe_2$<br>1T′ $MoTe_2$	650<br>356	184<br>127	7x10^{-7}<br>2.1x10^{-5}	-------<br>0.14	-------<br>-------	47
1T′ MoS_2 NR	79	36.2	8.80x10^{-6}	3.43	10.1	This work
1T′ $MoSe_2$ NR	327	48.3	4.32x10^{-8}	0.017	8.7	This work
1T′ $MoTe_2$ NR	441	76.6	3.41x10^{-7}	0.142	3.6	This work
1T′ WSe_2 NR	380	50.1	1.47x10^{-8}	0.006	8.2	This work

Supplementary Note 13. Calculation of TOF values for MX_2 NRs. We have considered only the edge active sites of MX_2 NR for the calculation of turnover frequencies (TOF) for a fair comparison with previously reported edge active MX_2 samples, where MoS_2 area coverage is not considered for the determination of exchange current density and TOF[27]. We have calculated the exchange current density per edge length (A/nm) from exchange current density (J_0, A/cm^2 obtained from equation S4 and Tafel plot) while considering that the NRs have an average length of 300 nm and a width of 40 nm. At the edges, HER activity takes place on the metallic sites, thus, based on the dimensions of the considered unit cell, we have calculated active sites as shown in Supplementary Fig. 15F. The calculated active site densities for MX_2 edges are given in Supplementary Table 2. This has been used to calculate the exchange current density per edge sites (A/sites), which is then multiplied by the site density of Pt (1.5 x 10^{15} sites/cm^2) for comparison[27]. TOF values were calculated from exchange current densities using the following equation (S7):

$$TOF\ (s^{-1}) = (Jo,\ A/cm^2)/[(1.5 \times 10^{15}\ sites/cm^2)(1.602 \times 10^{-19}\ C/e^-)\ (2\ e^-/H_2)] \qquad (S7)$$

Supplementary Table 2. HER activity of MX_2 NRs

1T′ NR Systems	Exchange current density (A/cm^2)	Exchange current density per edge length (A/nm)	Edge Active site per nm	Exchange current density per edge sites (A/sites)	Exchange current density (A/cm^2) normalized to Pt ($1.5x10^{15}$ sites $/cm^2$)	TOF (s^{-1})
MoS_2	$8.80x10^{-6}$	$3.52x10^{-18}$	3.2	$1.10x10^{-18}$	$1.65\ x10^{-3}$	3.43
$MoSe_2$	$4.32x10^{-8}$	$1.73x10^{-20}$	3.1	$5.57x10^{-21}$	$8.36\ x10^{-6}$	0.017
$MoTe_2$	$3.41x10^{-7}$	$1.36x10^{-19}$	3.0	$4.55x10^{-20}$	$6.82\ x10^{-5}$	0.142
WSe_2	$1.47x10^{-8}$	$5.88x10^{-21}$	3.1	$1.90x10^{-21}$	$2.85\ x10^{-6}$	0.006

References

1. Knirsch, K. C. *et al.* Basal-plane functionalization of chemically exfoliated molybdenum disulfide by diazonium salts. *ACS Nano* **9**, 6018-6030 (2015).
2. Voiry, D. *et al.* Covalent functionalization of monolayered transition metal dichalcogenides by phase engineering. *Nat. Chem.* **7**, 45 (2014).
3. El Garah, M. *et al.* MoS_2 nanosheets via electrochemical lithium-ion intercalation under ambient conditions. *FlatChem.* **9**, 33-39 (2018).
4. Jung, W. *et al.* Colloidal synthesis of single-layer MSe_2 (M= Mo, W) nanosheets via anisotropic solution-phase growth approach. *J. Am. Chem. Soc.* **137**, 7266-7269 (2015).
5. Huang, J.-K. *et al.* Large-area synthesis of highly crystalline WSe_2 monolayers and device applications. *ACS Nano* **8**, 923-930 (2013).
6. Sokolikova, M. S., Sherrell, P. C., Palczynski, P., Bemmer, V. L. & Mattevi, C. Direct solution-phase synthesis of 1T' WSe_2 nanosheets. *Nat. Commun.* **10**, 712 (2019).
7. Ambrosi, A., Sofer, Z. & Pumera, M. 2H → 1T phase transition and hydrogen evolution activity of MoS_2, $MoSe_2$, WS_2 and WSe_2 strongly depends on the MX_2 composition. *Chem. Commun.* **51**, 8450-8453 (2015).
8. Zhou, L. *et al.* Synthesis of high-quality large-area homogenous 1T′ $MoTe_2$ from chemical vapor deposition. *Adv. Mater.* **28**, 9526-9531 (2016).
9. Liu, M. *et al.* Synthesis of few-layer 1T′-$MoTe_2$ ultrathin nanosheets for high-performance pseudocapacitors. *J. Mater. Chem. A* **5**, 1035-1042 (2017).
10. Kresse, G. & Furthmuller, J. Efficiency of ab-initio total energy calculations for metals and semiconductors using a plane-wave basis set. *Comput. Mater. Science* **6**, 15-50 (1996).
11. Kresse, G. & Joubert, D. From ultrasoft pseudopotentials to the projector augmented-wave method. *Phys. Rev. B* **59**, 1758-1775 (1999).
12. Blöchl, P. E. Projector augmented-wave method. *Phys. Rev. B* **50**, 17953-17979 (1994).
13. Perdew, J. P., Ernzerhof, M. & Burke, K. Rationale for mixing exact exchange with density functional approximations. *J. Chem. Phys.* **105**, 9982-9985 (1996).
14. Grimme, S., Antony, J., Ehrlich, S. & Krieg, H. A consistent and accurate ab initio parametrization of density functional dispersion correction (DFT-D) for the 94 elements H-Pu. *J. Chem. Phys.* **132**, 154104 (2010).

15. Mathew, K., Sundararaman, R., Letchworth-Weaver, K., Arias, T. A. & Hennig, R. G. Implicit solvation model for density-functional study of nanocrystal surfaces and reaction pathways. *J. Chem. Phys.* **140**, 084106 (2014).
16. Monkhorst, H. J. & Pack, J. D. Special points for brillouin-zone integrations. *Phys. Rev. B* **13**, 5188-5192 (1976).
17. Skulason, E. *et al.* Density functional theory calculations for the hydrogen evolution reaction in an electrochemical double layer on the Pt (111) electrode. *Phys. Chem. Chem. Phys.* **9**, 3241-3250 (2007).
18. Rossmeisl, J., Logadottir, A. & Norskov, J. K. Electrolysis of water on (oxidized) metal surfaces. *Chem. Phys.* **319**, 178-184 (2005).
19. Norskov, J. K. *et al.* Origin of the overpotential for oxygen reduction at a fuel-cell cathode. *J. Phys. Chem. B* **108**, 17886-17892 (2004).
20. Peterson, A. A., Abild-Pedersen, F., Studt, F., Rossmeisl, J. & Nørskov, J. K. How copper catalyzes the electroreduction of carbon dioxide into hydrocarbon fuels. *Energy Environ. Sci.* **3**, 1311-1315 (2010).
21. Hansen, H. A., Rossmeisl, J. & Nørskov, J. K. Surface pourbaix diagrams and oxygen reduction activity of Pt, Ag and Ni (111) surfaces studied by DFT. *Phys. Chem. Chem. Phys.* **10**, 3722-3730 (2008).
22. Cho, S.-Y. *et al.* Highly enhanced gas adsorption properties in vertically aligned MoS_2 Layers. *ACS Nano* **9**, 9314-9321 (2015).
23. Yu, Y. *et al.* High phase-purity 1T′-MoS_2- and 1T′-$MoSe_2$-layered crystals. *Nat. Chem.* **10**, 638-643 (2018).
24. Gupta, U. *et al.* Characterization of few-layer 1T-$MoSe_2$ and its superior performance in the visible-light induced hydrogen evolution reaction. *APL Mater.* **2** (2014).
25. Zhou, L. *et al.* Large-area synthesis of high-quality uniform few-layer $MoTe_2$. *J. Am. Chem. Soc.* **137**, 11892-11895 (2015).
26. Song, Q. *et al.* Anomalous in-plane anisotropic Raman response of monoclinic semimetal 1 T′-$MoTe_2$. *Sci. Rep.* **7**, 1758 (2017).
27. Liu, D. *et al.* Atomically dispersed platinum supported on curved carbon supports for efficient electrocatalytic hydrogen evolution. *Nature Energy* **4**, 512-518 (2019).

28. Jaramillo, T. F. *et al.* Identification of active edge sites for electrochemical H_2 evolution from MoS_2 Nanocatalysts. *Science* **317**, 100 (2007).

29. Wang, H. *et al.* Electrochemical tuning of vertically aligned MoS_2 nanofilms and its application in improving hydrogen evolution reaction. *Proc.Natl. Acad. Sci.* **110**, 19701 (2013).

30. Kong, D. *et al.* Synthesis of MoS_2 and $MoSe_2$ films with vertically aligned layers. *Nano Let.* **13**, 1341-1347 (2013).

31. Voiry, D. *et al.* Conducting MoS_2 nanosheets as catalysts for hydrogen evolution reaction. *Nano Let.* **13**, 6222-6227 (2013).

32. Xie, J. *et al.* Controllable disorder engineering in oxygen-incorporated MoS_2 ultrathin nanosheets for efficient hydrogen evolution. *J. Am. Chem. Soc.* **135**, 17881-17888 (2013).

33. Peto, J. *et al.* Tapaszto, Spontaneous doping of the basal plane of MoS_2 single layers through oxygen substitution under ambient conditions. *Nat. Chem.* **10**, 1246-1251 (2018).

34. Oh, N. K. *et al.* Nafion-mediated liquid-phase exfoliation of transition metal dichalcogenides and direct application in hydrogen evolution reaction. *Chem. Mater.* **30**, 4658-4666 (2018).

35. Lukowski, M. A. *et al.* Enhanced hydrogen evolution catalysis from chemically exfoliated metallic MoS_2 nanosheets. *J. Am. Chem. Soc.* **135**, 10274-10277 (2013).

36. Li, Y. *et al.* Cracked monolayer 1T MoS_2 with abundant active sites for enhanced electrocatalytic hydrogen evolution. *Catal. Sci. Technol.* **7**, 718-724 (2017).

37. Geng, X. *et al.* Pure and stable metallic phase molybdenum disulfide nanosheets for hydrogen evolution reaction. *Nat. Commun.* **7**, 10672 (2016).

38. Yin, Y. *et al.* Contributions of phase, sulfur vacancies, and edges to the hydrogen evolution reaction catalytic activity of porous molybdenum disulfide nanosheets. *J. Am. Chem. Soc.* **138**, 7965-7972 (2016).

39. Eng, A. Y. S., Ambrosi, A., Sofer, Z., Šimek, P. & Pumera, M. Electrochemistry of transition metal dichalcogenides: Strong dependence on the metal-to-chalcogen composition and exfoliation method. *ACS Nano* **8**, 12185-12198 (2014).

40. Xie, J. *et al.* Defect-rich MoS_2 ultrathin nanosheets with additional active edge sites for enhanced electrocatalytic hydrogen evolution. *Adv. Mater.* **25**, 5807-5813 (2013).

41. Zhang, J. *et al.* Unveiling active sites for the hydrogen evolution reaction on monolayer MoS_2. *Adv. Mater.* **29**, 1701955-1701961 (2017).

42. Li, H. *et al.* Activating and optimizing MoS_2 basal planes for hydrogen evolution through the formation of strained sulphur vacancies. *Nature Materials* **15**, 48 (2015).

43. Zhang, Y. *et al.* Dendritic, Transferable, Strictly monolayer MoS_2 flakes synthesized on $SrTiO_3$ single crystals for efficient electrocatalytic applications. *ACS Nano* **8**, 8617-8624 (2014).

44. Zhang, J., Liu, S., Liang, H., Dong, R. & Feng, X. Hierarchical transition-metal dichalcogenide nanosheets for enhanced electrocatalytic hydrogen evolution. *Advanced Materials* **27**, 7426-7431 (2015).

45. Najafi, L. *et al.* Engineered $MoSe_2$-based heterostructures for efficient electrochemical hydrogen evolution reaction. *Advanced Energy Materials* **8**, 1703212 (2018).

46. McGlynn, J. C. *et al.* The rapid electrochemical activation of $MoTe_2$ for the hydrogen evolution reaction. *Nature communications* **10**, 1-9 (2019).

47. Seok, J. *et al.* Active hydrogen evolution through lattice distortion in metallic $MoTe_2$. *2D Materials* **4**, 025061 (2017).
